# METTL3-mediated m^6^A RNA methylation promotes the anti-tumour immunity of natural killer cells

**DOI:** 10.1038/s41467-021-25803-0

**Published:** 2021-09-17

**Authors:** Hao Song, Jiaxi Song, Ming Cheng, Meijuan Zheng, Tian Wang, Sha Tian, Richard A. Flavell, Shu Zhu, Hua-Bing Li, Chen Ding, Haiming Wei, Rui Sun, Hui Peng, Zhigang Tian

**Affiliations:** 1 Hefei National Laboratory for Physical Sciences at Microscale, the CAS Key Laboratory of Innate Immunity and Chronic Disease, School of Basic Medical Sciences, Division of Life Sciences and Medicine, University of Science and Technology of China, Hefei, Anhui 230027 China; 2 Institute of Immunology, University of Science and Technology of China, Hefei, Anhui 230027 China; 3 Department of Clinical Laboratory, The First Affiliated Hospital of Anhui Medical University, Hefei, Anhui 230022 China; 4 State Key Laboratory of Genetic Engineering, Human Phenome Institute, Institutes of Biomedical Sciences, School of Life Sciences, Zhongshan Hospital, Fudan University, Shanghai, 200032 China; 5 Department of Immunobiology, Yale University School of Medicine, New Haven, CT 06520 USA; 6 Howard Hughes Medical Institute, Chevy Chase, MD 20815 USA; 7 Shanghai Institute of Immunology, State Key Laboratory of Oncogenes and Related Genes, Shanghai Jiao Tong University School of Medicine, Shanghai, 200025 China; 8 Research Unit of NK Cell Study, Chinese Academy of Medical Sciences, Hefei, Anhui 230027 China

**Keywords:** Immunosurveillance, Interleukins, NK cells

## Abstract

Natural killer (NK) cells exert critical roles in anti-tumor immunity but how their functions are regulated by epitranscriptional modification (e.g., N^6^-methyladenosine (m^6^A) methylation) is unclear. Here we report decreased expression of the m^6^A “writer” METTL3 in tumor-infiltrating NK cells, and a positive correlation between protein expression levels of METTL3 and effector molecules in NK cells. Deletion of *Mettl3* in NK cells alters the homeostasis of NK cells and inhibits NK cell infiltration and function in the tumor microenvironment, leading to accelerated tumor development and shortened survival in mice. The gene encoding SHP-2 is m^6^A modified, and its protein expression is decreased in METTL3-deficient NK cells. Reduced SHP-2 activity renders NK cells hyporesponsive to IL-15, which is associated with suppressed activation of the AKT and MAPK signaling pathway in METTL3-deficient NK cells. These findings show that m^6^A methylation safeguards the homeostasis and tumor immunosurveillance function of NK cells.

## Abstract (web summary)

Post transcriptional modifications to RNA may be important in the function of NK cells but this is not fully known. Here the authors show that m^6^A-methyltransferase METTL3 deletion in NK cells leads to reduced function and protection against tumour challenge through suppressing response to IL-15.

## Introduction

As a central component of the innate immune system, natural killer (NK) cells are important in tumor surveillance^1–4^. Unlike adaptive lymphocytes, activation of NK cells is determined by the integration of signals from germline-encoded activating and inhibitory receptors^5,6^. Upon activation, NK cells can kill target malignant cells via the release of lytic granules containing perforin and granzymes, and secrete cytokines with potent anti-tumor activities^7–9^. Meanwhile, NK cells can also mediate cytotoxicity via death ligands, such as Fas ligand (FasL) and tumor necrosis factor-related apoptosis-inducing ligand (TRAIL), which induce apoptosis of target cells^10–12^.

The effector functions of NK cells are also regulated by soluble molecules. Among these, interleukin (IL)-15 is a critical cytokine controlling the survival, proliferation, and activation of NK cells through the IL15 receptor (IL-15R) complex, which consists of the α, β (CD122), and common γ (γ_c_, CD132) chains^13,14^. IL-15Rα is expressed widely on many cell types and binds to IL-15 with high affinity to form a heterodimer that presents IL-15 *in trans* to IL-15Rβ-γ_c_-expressing cells^15^. Engagement of IL-15Rβ-γ_c_ induces activation of JAK1/3-STAT5, Ras-MAPK-ERK, and PI3K-AKT-mTOR pathways, thereby exerting diverse effects on the biology of NK cells^16–19^. However, the regulation of IL-15R downstream signaling pathways is incompletely understood.

In recent years, *N*^6^-methyladenosine (m^6^A) modification has drawn much attention due to its prevalence and importance in eukaryotes^20^. There are three independent components related to m^6^A modification: “writers”, “erasers”, and “readers” ^20^. Methyltransferase-like (METTL) 3, METTL14 and Wilms' tumor 1-associated protein (WTAP) belong to “writers”, and form the m^6^A methyltransferase complex, which installs m^6^A on target messenger (m)RNA^21^. Although both METTL3 and METTL14 contain methyltransferase domains; studies show that METTL3 is the catalytically active subunit, while METTL14 functions as an RNA-binding platform^22^. m^6^A modification regulates the splicing, translation, and stability of mRNA, thereby influencing the differentiation, proliferation, and apoptosis of cells^23–26^. For instance, m^6^A modification is required for the specification and differentiation of hematopoietic stem cells^27–30^. Studies have shown that m^6^A methylation is involved in innate immune cell-mediated antiviral immunity^31–34^, and has emerged as a marker of circular RNAs for self-avoidance of activation of retinoic acid-inducible gene-1^35^. For adaptive immunity, it has been shown that m^6^A methylation supports the proliferation and differentiation of CD4^+^ T cells, and controls the immune-suppressive functions of T regulatory cells^36,37^. m^6^A methylation also impedes the cross-presentation capacity of dendritic cells via YTH domain-containing family protein-1 (YTHDC1), an m^6^A reader protein, thereby inhibiting the CD8^+^ T cell-mediated antitumor response^38^. Paradoxically, METTL3-mediated m^6^A methylation promotes dendritic cell activation and the ability to prime T cells^39^. Although the role of m^6^A methylation in the immune system has been increasingly appreciated, its influence upon NK cells has only recently started to be recognized. A previous study reported that m^6^A “reader” YTHDF2 promotes NK cell effector functions^40^. However, the function of m^6^A “writers” and upstream environmental factors regulating m^6^A methylation in NK cells is currently unknown.

In this study, we find that METTL3 expression is positively correlated with the m^6^A levels and effector functions of NK cells. Mice conditionally deficient for METTL3 in NK cells have aggravated tumor progression, accompanied by a reduced number and impaired effector functions of NK cells. Further analyses show that METTL3-mediated m^6^A methylation safeguards signaling pathways downstream of IL-15R, thereby regulating NK cell responsiveness to IL-15 in the tumor microenvironment (TME).

## Results

### Reduced expression of METTL3 in tumor-infiltrating NK cells

**Fig. 1:**
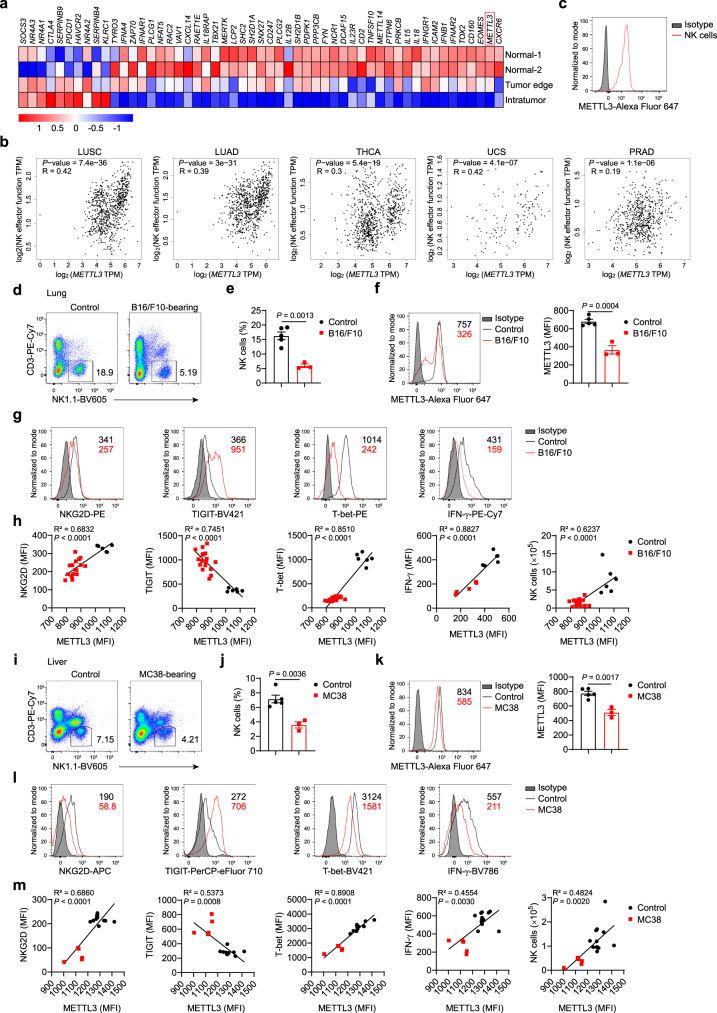
METTL3 is positively correlated with effector functions of NK cells. **a** Heatmap showing the NK cell effector function-related genes in tumor-infiltrating NK cells compared with those in normal liver NK cells from a published dataset (GSE120123). **b** Correlation between *METTL3* TPM (transcripts per kilobase of exon model per million mapped reads) and module TPM of NK effector function (*NCR1*, *EOMES*, *TBX21*, *CD69*, *DNAM1*, *KLRK1*, *PERF*, *IFNG*, and *GZMB*) in lung squamous cell carcinoma (LUSC), lung adenocarcinoma (LUAD), thyroid carcinoma (THCA), uterine carcinosarcoma (UCS), and prostate adenocarcinoma (PRAD) tissues. Spearman’s correlation coefficient (R) and *P* values are shown. **c** A representative histogram showing METTL3 expression in peripheral blood NK cells from a healthy human. **d**–**h** WT C57BL/6 mice were harvested for analyses of lung NK cells 3–4 weeks after intravenous injection of 5 × 10^5^ B16/F10 cells. **d** Representative plots showing expression of NK1.1 and CD3 in lungs. **e** Percentages of NK cells from control (*n* = 5) or B16/F10-bearing (*n* = 3) mice. **f** A representative histogram (left) and MFI (right) of METTL3 expression in NK cells from control (*n* = 5) or B16/F10-bearing (*n* = 3) mice. **g** Representative histograms of indicated-molecule expression in NK cells from control or B16/F10-bearing mice. For detection of IFN-γ, lung mononuclear cells were isolated and stimulated with phorbol 12-myristate 13-acetate (PMA) and ionomycin for 4 h for flow-cytometric analyses. **h** Correlation between the MFI of METTL3 expression and MFI of the indicated molecule expression in NK cells, or NK cell numbers (*n* = 6 for the control group; *n* = 5 (IFN-γ) or 17 (other molecules) for B16/F10 group). **i**–**m** WT C57BL/6 mice were harvested for analyses of liver NK cells 3–4 weeks after intrasplenic injection of 2 × 10^5^ MC38 cells. **i** Representative plots showing expression of NK1.1 and CD3 in livers. **j** Percentages of liver NK cells from control (*n* = 5) or MC38-bearing (*n* = 3) mice. **k** A representative histogram (left) and MFI (right) of METTL3 expression in liver NK cells from control (*n* = 5) or MC38-bearing (*n* = 3) mice. **l** Representative histograms of indicated-molecule expression in liver NK cells from control or MC38-bearing mice. For detection of IFN-γ, liver mononuclear cells were isolated and stimulated with PMA and ionomycin for 4 h for flow-cytometric analyses. **m** Correlation between the MFI of METTL3 expression and MFI of the indicated molecule expression in NK cells, or NK cell numbers (*n* = 12 for the control group; *n* = 5 for MC38 group). Each symbol represents an individual mouse (**e**, **f**, **h**, **j**, **k**, **m**). Data are the mean ± SEM (unpaired two-tailed *t* test (**e**, **f**, **j**, **k**), two-tailed Pearson correlation-test (**h**, **m**)). Source data are provided as a Source Data file. Data represent at least two independent experiments (**c**, **d**–**m**).

As a pivotal antitumor effector of the innate immune system, NK cells hold great promise in cancer immunotherapy^41^. However, the complicated TME can hinder NK cell-mediated effective control of tumors. To better understand the characteristics of NK cells in the TME, we analyzed microarray data of tumor-infiltrating or tumor-edge NK cells from patients with hepatocellular carcinoma (HCC), or NK cells from normal liver tissue^42^. We found that numerous NK cell effector function-related genes, such as *TBX21*, *FYN*, *NCR1*, *SH2D1A*, *SH2D1B*, *PTPN6*, and *IL18RAP*, were downregulated in tumor-infiltrating NK cells (Fig. 1a). Conversely, expression of the genes associated with NK cell exhaustion, such as *PDCD1*, *CTLA4*, *KLRC1*, *NR4A1*, and *SOCS3*, was upregulated in tumor-infiltrating NK cells (Fig. 1a). Similarly, NK cells from ascites of human ovarian cancer patients also downregulated NK cell effector function-related genes and upregulated NK cell exhaustion-related genes, compared to peripheral blood NK cells from healthy donors (Supplementary Fig. 1A). Notably, we observed that m^6^A “writers” *METTL3* and *METTL14* mRNA levels were respectively decreased by ~75% and ~49% in tumor-infiltrating NK cells of HCC patients (Fig. 1a). Besides, there was a reduction (~34%) of *METTL3* mRNA expression but unchanged *METTL14* mRNA expression in ascites NK cells of ovarian cancer patients (Supplementary Fig. 1A). Moreover, using Gene Expression Profiling Interactive Analysis (GEPIA) online tools^43^, we found that *METTL3* mRNA expression was positively correlated with the module of the effector function of NK cells in different human tumors based on the database of The Cancer Genome Atlas (TCGA) and Genotype-Tissue Expression (GTEx) (Fig. 1b). These results suggest that METTL3 might contribute to the effector function of NK cells.

We then measured METTL3 protein expression and found that NK cells from the peripheral blood of healthy humans were mostly METTL3-positive (Fig. 1c and Supplementary Fig. 1B). To further confirm the relationship between METTL3 expression and the effector functions of NK cells, we used a mouse model of pulmonary metastases of B16/F10 melanoma. Three to four weeks after tumor inoculation (a late stage of tumor growth, Supplementary Fig. 1C), we found a dramatic reduction of the percentage of NK cells in the lungs (Fig. 1d, e and Supplementary Fig. 1D). In agreement with that found in human NK cells, mouse NK cells at a steady state displayed predominantly positive expression of METTL3 protein (Fig. 1f). Surprisingly, METTL3 protein expression in NK cells from tumor-bearing mice was significantly decreased (Fig. 1f), and the suppressed METTL3 protein expression was increasingly evident with tumor progression (Supplementary Fig. 1E). Interestingly, METTL3 protein expression exhibited a positive correlation with the number of NK cells in the lungs, thereby suggesting that METTL3 might be important for NK cell maintenance in the TME (Fig. 1h). Furthermore, expression of NKG2D, T-bet, and interferon (IFN)-γ was positively correlated with METTL3 protein expression (Fig. 1g, h). In contrast, there is a negative correlation between protein expression levels of the checkpoint receptor TIGIT and METTL3 in NK cells (Fig. 1g, h). Similar results were observed in a model of colorectal cancer (CRC) liver metastases generated by intrasplenic injection of MC38 tumor cells (Fig. 1i–m and Supplementary Fig. 1F).

The TME is enriched with certain immunosuppressive and inflammatory cytokines^44^. To investigate the factors contributing to the suppression of METTL3 expression in the TME, we stimulated splenocytes with different cytokines in vitro. We found that IL-17F, IL-18, and IL-27 had little impact on METTL3 protein expression in NK cells, while IL-15 and IL-10 could increase METTL3 protein expression; only TGF-β treatment significantly decreased METTL3 protein expression (Supplementary Fig. 1G). Furthermore, only the protein level of METTL3, but not other m^6^A methylation-related proteins, such as WTAP, FTO, ALKBH5, YTHDC1, YTHDC2, and YTHDF2, was obviously reduced in NK cells when treated with TGF-β (Supplementary Fig. 1H). To confirm the TME-mediated decreased METTL3 protein expression in NK cells, we cocultured NK cells with MC38 tumor cells and consistently found that MC38 cells could decrease METTL3 protein expression in NK cells. Importantly, the presence of the TGF-βRI inhibitor GW788388 in the culture system counterbalanced the MC38 cell-mediated inhibition of METTL3 protein expression in NK cells, but the SMAD3 inhibitor SIS3 failed to (Supplementary Fig. 1I). These results suggest that TGF-β downregulates METTL3 protein expression in NK cells independently of SMAD3.

Previous studies suggest that low levels of METTL3 expression are adequate for preserving mRNA methylations in some cell types^45^. However, we found that the decreased METTL3 protein expression in TGF-β-treated NK cells reduced m^6^A levels by ~22% (Supplementary Fig. 1J). Moreover, METTL3 protein expression reduced at a moderate extent in NK92 cells (a human NK cell line) using shRNA also resulted in a reduction (~49%) of m^6^A levels (Supplementary Fig. 2A, B). Consistent with the observations of tumor-infiltrating NK cells, downregulation of METTL3 protein level in NK92 cells induced decreased expression of activation receptors, such as CD69, NKp30, and NKG2D, the transcription factor T-bet, and IFN-γ (Supplementary Fig. 2C, D). Altogether, these results collectively indicate that reduced METTL3 protein expression induced by TGF-β in the TME contributes to decreased m^6^A methylation and impaired effector functions of NK cells.

### The tumoricidal activity of NK cells is dependent upon METTL3

**Fig. 2:**
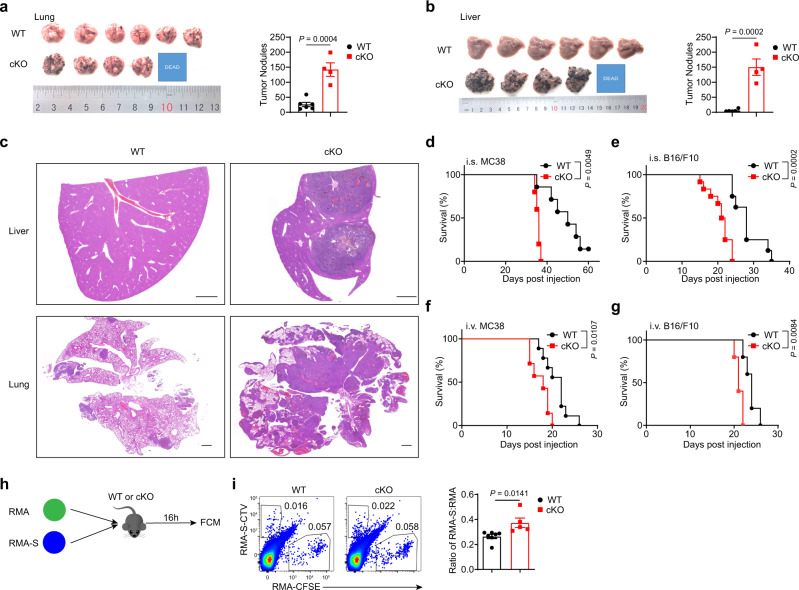
*Mettl3* deletion in NK cells fails to control tumor development. **a** Imaging of lungs (left) and tumor nodules in lungs (right) from WT (*n* = 6) or cKO (*n* = 4) mice 16 days after intravenous injection of 5 × 10^5^ B16/F10 cells. **b** Imaging of livers (left), and tumor nodules in livers (right) from WT (*n* = 6) or cKO (*n* = 4) mice 16 days after intravenous injection of 5 × 10^5^ B16/F10 cells. **c** Representative hematoxylin and eosin (H&E) staining images of the liver (top) and lung samples (bottom) from WT mice or cKO mice after intravenous injection of 2 × 10^5^ B16/F10 cells. Scale bars represent 1 mm. **d** Survival of WT mice (*n* = 7) and cKO mice (*n* = 5) after intrasplenic injection of 2 × 10^5^ MC38 cells. **e** Survival of WT mice (*n* = 8) and cKO mice (*n* = 12) after intrasplenic injection of 2 × 10^5^ B16/F10 cells. **f** Survival of WT mice (*n* = 9) and cKO mice (*n* = 7) after intravenous injection of 5 × 10^5^ MC38 cells. **g** Survival of WT mice (*n* = 5) and cKO mice (*n* = 5) after intravenous injection of 2.5 × 10^5^ B16/F10 cells. **h** WT mice or cKO mice were co-transferred with 5 × 10^6^ CFSE-labeled RMA cells and 1.5 × 10^7^ CTV labeled RMA-S cells, and splenocytes were harvested 16 h later. **i** Representative plots (left) showing expression of CTV and CFSE in the spleen, and a statistical graph showing the ratio of RMA-S cells: RMA cells (*n* = 7) or cKO (*n* = 5) mice in (**i**). Each symbol represents an individual mouse (**a**, **b**, **d**, **e**, **f**, **g**, **i**). Data are the mean ± SEM (unpaired two-tailed *t* test (**a**, **b**, **i**) or two-tailed log-rank (Mantel–Cox) test (**d**–**g**)). Source data are provided as a Source Data file. Data represent at least two independent experiments (**a**–**d**, **g**-**i**) or are pooled from two independent experiments (**e**, **f**).

To investigate if METTL3 is required for NK cell-mediated anti-tumor immunity, we generated *Mettl3*^fl/fl^-*Ncr1*^Cre/*+*^ (cKO) mice, which harbored a selective deficiency of METTL3 expression at the mRNA or protein level in NK cells, with no change in METTL3 protein expression of T and B cells (Supplementary Fig. 3A–D). After intravenous injection of B16/F10 cells, cKO mice had significantly more lung tumor nodules than WT mice, displaying more progressive pulmonary metastases (Fig. 2a, c). Moreover, metastatic foci were present abundantly in the livers of cKO mice, but rarely in WT mouse livers (Fig. 2b, c). In the CRC liver metastasis model induced by intrasplenic injection of MC38 cells, we found that cKO mice also developed more liver metastases compared with that in WT controls (Supplementary Fig. 3E, F). In a subcutaneous tumor model, cKO mice bearing MC38 tumors also exhibited accelerated tumor growth (Supplementary Fig. 3G). In agreement with these pathologic changes, the overall survival of cKO mice was shortened significantly in different tumor models generated by intravenous, intrasplenic, or subcutaneous injection of MC38 or B16/F10 cells (Fig. 2d–g, and Supplementary Fig. 3H). These results demonstrate that METTL3 deficiency in NK cells results in increased metastases and shortened survival.

To further confirm the role of METTL3 in NK cell-mediated anti-tumor immunity, we depleted NK cells in WT mice or cKO mice by using anti-asialo-GM1 (ASGM1) or anti-NK1.1, followed by intrasplenic injection of MC38 cells (Supplementary Fig. 4A, C). Consequently, NK cell depletion accelerated tumor progression in WT mice, as evidenced by reduced survival in NK-cell-depleted mice, confirming that NK cells were required for tumor immunosurveillance (Supplementary Fig. 4B, D). Moreover, there was no significant difference in overall survival between cKO mice without NK cell depletion treatment and NK cell-depleted WT mice (Supplementary Fig. 4B, D), suggesting that METTL3 deficiency largely abrogated the NK cell immunosurveillance of cancer. Furthermore, intravenous injection of a mixture of NK cell-sensitive target RMA-S cells and NK cell-resistant target RMA cells showed that METTL3-deficient NK cells could not kill RMA-S cells efficiently compared with METTL3-sufficient NK cells (Fig. 2h, i). In addition, METTL3-deficient NK cells exhibited lower cytotoxic activity against YAC-1 target cells in vitro than WT NK cells (Supplementary Fig. 4E). Collectively, these data indicate that METTL3 is indispensable for NK cell-mediated antitumor immunity.

### METTL3 deficiency disturbs the homeostasis of NK cells

**Fig. 3:**
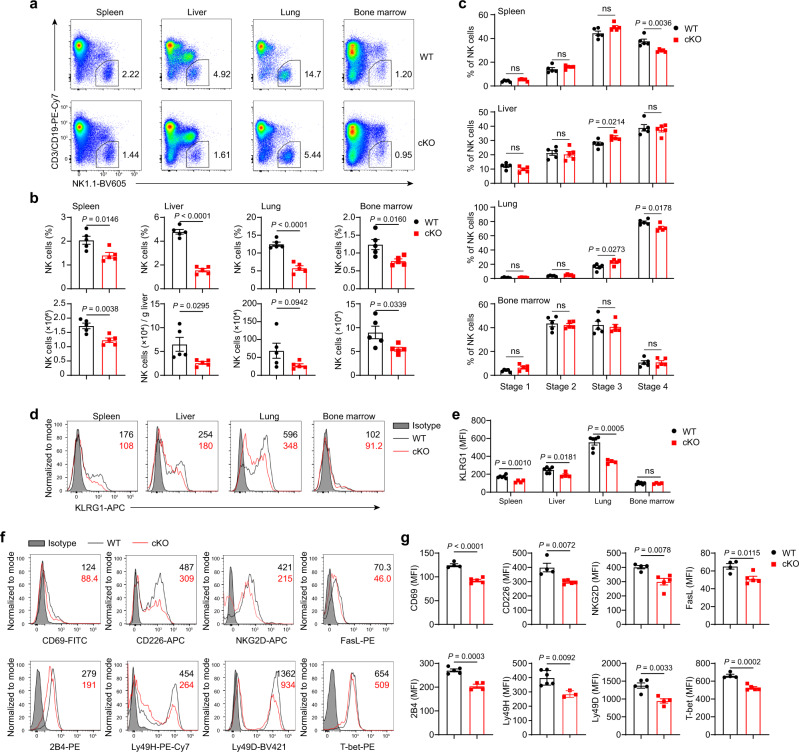
METTL3 deficiency disturbs NK cell homeostasis at a steady state. **a** Representative plots showing expression of NK1.1 and CD3/CD19 in mononuclear cells from the indicated organs of WT and cKO mice. **b** Percentages and absolute numbers of NK cells in the indicated organs from WT and cKO mice (*n* = 5/group). **c** Percentages of NK cells at different stages in the indicated organs of WT and cKO mice (*n* = 5/group). (Stage 1: CD27^−^ CD11b^−^; stage 2: CD27^+^ CD11b^−^; stage 3: CD27^+^ CD11b^+^; stage 4: CD27^−^ CD11b^+^). **d**, **e** Representative histograms (**d**) and MFI (**e**) of KLRG1 expression in NK cells from the indicated organs of WT (*n* = 6) and cKO (*n* = 4) mice. **f**, **g** Representative histograms (**f**) and MFI (**g**) of expression of the indicated molecules in splenic NK cells from WT (*n* = 5 for 2B4 and Ly49D, *n* = 6 for Ly49H, and *n* = 4 for other molecules) and cKO mice (*n* = 3 for Ly49H, *n* = 4 for 2B4 and Ly49D, and *n* = 5 for other molecules). Each symbol represents an individual mouse (**b**, **c**, **e**, **g**). Data are the mean ± SEM (ns, not significant; unpaired two-tailed *t*-test). Source data are provided as a Source Data file. Data represent two (**d**, **e**) or at least three independent experiments (**a**–**c**, **f**, **g**).

The finding that *Mettl3* deletion in NK cells induced accelerated tumor growth prompted investigation of whether METTL3 was required for NK cell maintenance. Compared with WT controls, cKO mice showed a reduced percentage and an absolute number of NK cells in the spleen, liver, lung, and bone marrow (Fig. 3a, b). We then wondered whether reduced NK cell numbers in cKO mice reflected the altered proliferation or survival of NK cells. However, we found no significant difference in expression of the proliferation marker Ki67 and the anti-apoptotic factor Bcl2, or in frequency of Annexin V^+^ apoptotic cells between NK cells of cKO mice and WT mice (Supplementary Fig. 5A–C). Although previous studies show that METTL3-mediated m^6^A methylation regulates cell cycle^46,47^, we observed unaltered cell cycle progression in METTL3-deficient NK cells (Supplementary Fig. 5D, E).

We further questioned whether NK cell development was altered by METTL3 deficiency. However, the percentage and number of NK precursors (NKPs) in the bone marrow were unchanged in cKO mice (Supplementary Fig. 5F, G). By analyzing the stages of NK cell maturation based on cell surface expression of CD27 and CD11b, we found a lower frequency of stage 4 (CD11b^+^ CD27^−^) NK cells in the spleen and lung of cKO mice, but the normal frequency in the liver and bone marrow (Fig. 3c). The inhibitory killer cell lectin-like receptor G1 (KLRG1), a marker of terminally differentiated NK cells^48^, was downregulated in peripheral organs (Fig. 3d, e). Moreover, expression of the activating molecules CD69, CD226, NKG2D, 2B4, Ly49H, and Ly49D was simultaneously decreased in splenic NK cells from cKO mice (Fig. 3f, g). In addition, METTL3-deficient NK cells had lower expression of the apoptosis-inducing molecule FasL than those in WT mice (Fig. 3f, g). Furthermore, *Mettl3* deletion diminished expression of the transcription factor T-bet in splenic NK cells (Fig. 3f, g). Similarly, METTL3 deficiency also resulted in reduced expression of effector function-related molecules in NK cells from the liver and lung (Supplementary Fig. 6A, B). Therefore, these data reveal that METTL3 is pivotal for sustaining NK cell numbers, terminal maturation, and expression of effector function-associated molecules.

### METTL3 supports the infiltration and function of NK cells in the TME

**Fig. 4:**
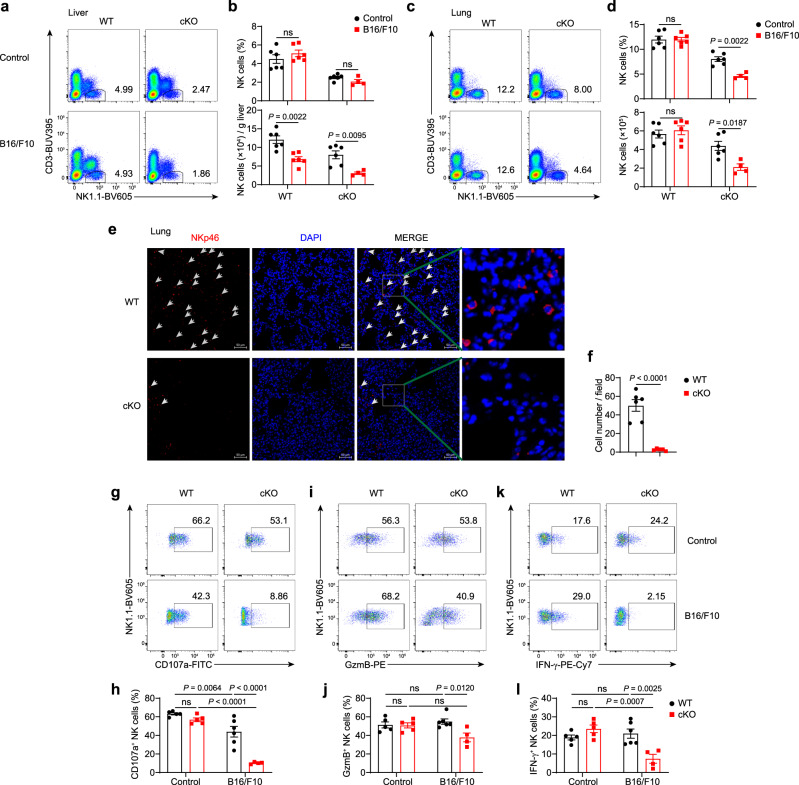
METTL3 supports NK cells accumulation and effector function in the TME. **a**–**d** WT mice and cKO mice were harvested for flow-cytometric analyses 16 days after intravenous injection with 5 × 10^5^ B16/F10 cells or PBS. **a**, **c** Representative plots showing expression of NK1.1 and CD3 in the liver (**a**) or lung (**c**) mononuclear cells from WT and cKO mice. **b**, **d** Percentages or absolute numbers of the liver (**b**) or lung (**d**) NK cells from WT mice (*n* = 6/group) and cKO mice (*n* = 6 for the control group; *n* = 4 for B16/F10 group). **e** Immunofluorescence staining of lungs with anti-NKp46 (red) and DAPI (blue) from WT or cKO mice 25 days after intravenous injection with 2 × 10^5^ B16/F10 cells. Scale bar, 50 μm. The arrows indicate NK cells. **f** NKp46-positive cell numbers per field based on (**e**) (*n* = 6 for WT group; *n* = 5 for cKO group). **g**–**l** WT mice and cKO mice were harvested for flow-cytometric analyses 23 days after intravenous injection with 2 × 10^5^ B16/F10 cells or PBS. Liver mononuclear cells were isolated and stimulated with PMA and ionomycin for 4 h for flow-cytometric analyses. Representative plots and percentages for the expression of CD107a (**g**, **h**), GzmB (**i**, **j**), and IFN-γ (**k**, **l**) expression among liver NK cells from WT (*n* = 5 for the control group; *n* = 6 for B16/F10 group) and cKO mice (*n* = 5 for the control group; *n* = 4 for B16/F10 group). Each symbol represents an individual mouse (**b**, **d**, **h**, **j**, **l**) or individual field of the microscope (**f**). Data are the mean ± SEM (ns, not significant; unpaired two-tailed *t* test (**f**) or one-way ANOVA (**b**, **d**, **h**, **j**, **l**)). Source data are provided as a Source Data file. Data represent at least two independent experiments (**a**–**l**).

Next, we sought to explore how METTL3 influences the antitumor responses of NK cells. In contrast to the reduction of lung NK cells in a late-stage metastatic melanoma model (Fig. 1d, e), NK cell percentages and numbers in the lung and liver were not significantly changed at an earlier stage of tumor development in WT mice (Fig. 4a–d); however, tumor-bearing cKO mice had decreased liver and lung NK cells during this period, compared to those without tumor implantation (Fig. 4a–d). METTL3 deficiency thus caused a more profound reduction of NK cells in the TME than at a steady state. Immunofluorescence analyses further confirmed that there were fewer tumor-infiltrating NK cells in the lung or liver of cKO mice than those of WT mice (Fig. 4e, f, and Supplementary Fig. 7A, B). Furthermore, the apoptosis and proliferation of NK cells were unaltered in tumor-bearing cKO mice and thus were not responsible for reduced NK cells (Supplementary Fig. 7C, D).

The TME also induced downregulation of activation molecules and the transcription factor Eomes, and upregulation of the inhibitory receptor TIGIT in METTL3-deficient NK cells, at a more evident extent than in METTL3-sufficient NK cells (Supplementary Fig. 7E). By comparing expression of effector molecules in METTL3-sufficient and METTL3-deficient NK cells, we found no significant differences in CD107a, granzyme B (GzmB), and IFN-γ protein expression between METTL3-deficient and -sufficient NK cells at a steady-state after phorbol 12-myristate 13-acetate (PMA) plus ionomycin stimulation (Fig. 4g–l). In contrast, METTL3-deficient NK cells from tumor-bearing mice exhibited remarkably decreased protein expression of CD107a, GzmB and IFN-γ than METTL3-sufficient NK cells (Fig. 4g–l), indicating METTL3 deficiency-mediated inhibition of cytotoxicity and cytokine production of NK cells in the TME. Thus, these data suggest dampened ability of METTL3-deficient NK cells to respond to stimuli present in the TME.

### METTL3 regulates the responsiveness of NK cells to IL-15

**Fig. 5:**
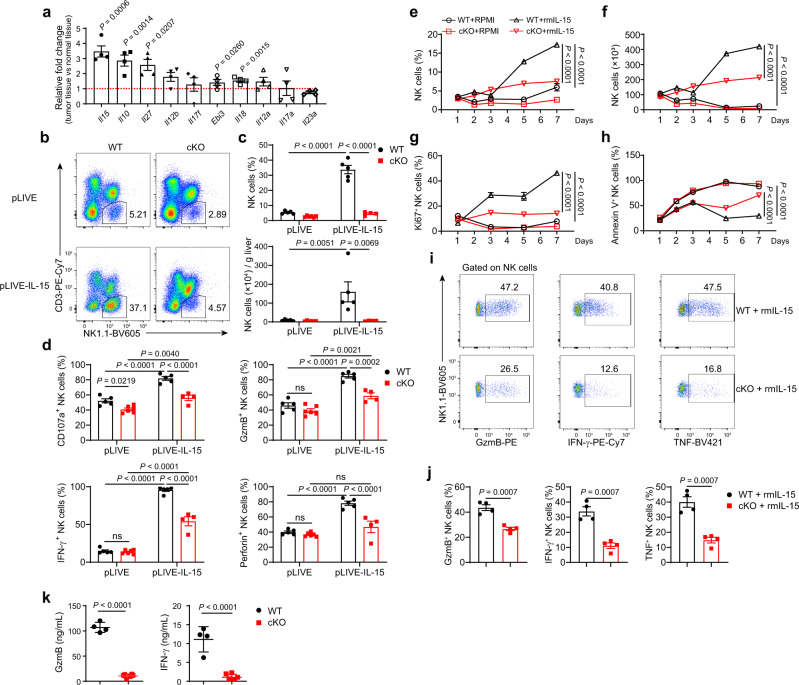
METTL3-deficient NK cells are hyporesponsive to IL-15 stimulation. **a** Fold changes of cytokine expression in tumor-bearing liver tissue (*n* = 4) from WT mice injected with 2 × 10^5^ MC38 cells into the spleen 22 days previously, compared with normal liver tissue (*n* = 4), were determined by RT-qPCR, normalized to *β-actin* expression. **b**–**d** WT (*n* = 5/group) or cKO mice (*n* = 6 for pLIVE group; *n* = 4 for the pLIVE-IL-15 group) that received a hydrodynamic injection of pLIVE-IL-15 or pLIVE control plasmid 1 month previously were harvested for flow-cytometric analyses. **b** Representative plots showing expression of NK1.1 and CD3 in liver mononuclear cells from WT and cKO mice. **c** Percentages or absolute numbers of liver NK cells from WT and cKO mice. **d** Percentages of CD107a-, GzmB-, IFN-γ-, and Perforin-positive NK cells among total NK cells in splenocytes 4 h after stimulated with PMA and ionomycin. **e**–**h** Splenocytes from WT or cKO mice were cultured with the presence or absence of rmIL-15 (100 ng/mL) for the indicated times, and then analyzed by flow cytometry. Percentages (**e**) and absolute numbers (**f**) of NK cells among splenocytes are shown (*n* = 4/group). **g** Percentages of Ki67-positive cells among NK cells (*n* = 3/group). **h** Percentages of Annexin V-positive cells among NK cells (*n* = 4/group). **i**, **j** Splenocytes from WT mice or cKO mice were stimulated with rmIL-15 (10 ng/mL) for 7 days, and then collected for stimulation with PMA and ionomycin, followed by flow cytometric analyses. Representative plots (**i**) and percentages (**j**) of expression of GzmB, IFN-γ, and TNF in NK cells from WT and cKO mice (*n* = 4/group). **k** Protein concentration of GzmB and IFN-γ in the supernatant of cultured NK cells in the condition of rmIL-15 (50 ng/mL) for 9 days from WT (*n* = 4) or cKO (*n* = 6) mice. Each symbol represents an individual mouse (**c**, **d**) or an individual well of a cell culture plate (**e**–**h**, **j**, **k**). Data are the mean ± SEM (ns, not significant; unpaired two-tailed *t* test (**a**, **j**, **k**) or one-way ANOVA (**c**, **d**) or two-way ANOVA (**e**–**h**)). Source data are provided as a Source Data file. Data represent at least two independent experiments (**a**–**k**).

**Fig. 6:**
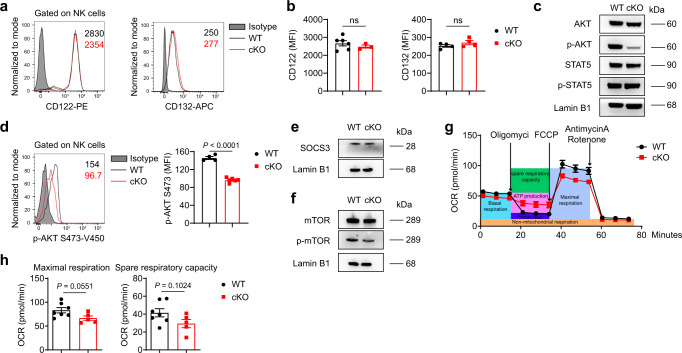
The IL-15-AKT pathway is impaired in METTL3-deficient NK cells. **a**, **b** Representative histograms (**a**) and MFI (**b**) of CD122 and CD132 expression in splenic NK cells from WT (*n* = 6 for CD122, *n* = 4 for CD132) and cKO (*n* = 3 for CD122, *n* = 4 for CD132) mice. **c**, **e**, **f** Immunoblotting of indicated molecules in splenic NK cells upon stimulation with rmIL-15 (50 ng/mL) for 1 h. The NK cells were sorted from WT and cKO mice that received hydrodynamic injection of the pLIVE-IL-15 plasmid or pLIVE control plasmid 1–2 months previously. **d** Representative histogram (left) and MFI (right) of p-AKT (S473) expression in splenic NK cells upon stimulation with rmIL-15 (50 ng/mL) for 1 h (*n* = 4 for WT group; *n* = 6 for cKO group). **g**, **h** Splenic NK cells from WT or cKO mice were cultured with rmIL-15 overnight (10 ng/mL), and then the indicated compounds were added into each well at the indicated times. **g** The oxygen consumption rate was measured for NK cells. **h** Statistical graphs showing the maximal respiration and spare respiratory capacity of NK cells (*n* = 7 for WT group; *n* = 5 for cKO group). Each symbol represents an individual mouse (**b**, **d**) or an individual well of a cell culture plate (**h**). Data are the mean ± SEM (ns, not significant; unpaired two-tailed *t* test (**b**, **d**, **h**)). Source data are provided as a Source Data file. Data represent at least two independent experiments (**a**–**h**).

IL-15 signaling is critical for the development, proliferation, and function of NK cells^19,49,50^. The expression of several cytokines associated with the effector functions of NK cells was increased in the TME (Fig. 5a). The increase in *Il15* expression was particularly significant (Fig. 5a). Considering the reduced number of NK cells and suppressed effector functions of tumor-infiltrating NK cells in cKO mice, we wondered if METTL3-deficient NK cells were defective in their response to IL-15. To test our hypothesis, we overexpressed IL-15 in vivo via hydrodynamic injection of the pLIVE-IL-15 plasmid into WT mice or cKO mice (Supplementary Fig. 8A). Consistent with the previous findings that IL-15 increased NK cell numbers^51^, we noted a six-fold increase in proportion and a more-than-tenfold increase in the number of liver NK cells after pLIVE-IL-15 treatment (Fig. 5b, c). However, in cKO mice, the percentages or numbers of NK cells exhibited no more than the twofold increase (Fig. 5b, c). Similarly, the number of NK cells from the spleen and lung of cKO mice failed to increase vigorously in response to IL-15 stimulation in vivo (Supplementary Fig. 8B, C). In contrast to moderately changed maturation of METTL3-deficient NK cells at steady state (Fig. 3c), IL-15 overexpression led to more profound differences in maturation status between cKO and WT NK cells, with a less mature phenotype of NK cells in cKO mice (Supplementary Fig. 8D–F). Moreover, NK cells from IL-15-overexpressing cKO mice showed a lower expression of cytotoxic molecules and IFN-γ upon stimulation with PMA plus ionomycin than that of their WT counterparts, despite no significant differences between NK cells from control WT mice and cKO mice (Fig. 5d, Supplementary Fig. 8G, H). Furthermore, stimulation of splenocytes with recombinant mouse IL-15 (rmIL-15) in vitro induced a significant increase in the percentages and numbers of NK cells from WT mice, whereas the percentages and numbers of NK cells from cKO mice increased only slightly (Fig. 5e, f). In addition, METTL3-deficient NK cells displayed inferior proliferation and survival (Fig. 5g, h, and Supplementary Fig. 8I) and defective ability for expressing GzmB, IFN-γ and tumor necrosis factor (TNF) (Fig. 5i–k) in response to IL-15, as compared with NK cells from WT mice. Thus, these results suggest that METTL3 deficiency impairs the responsiveness of NK cells to IL-15. Next, we sought to investigate the molecular mechanisms leading to impaired IL-15 response of METTL3-deficient NK cells. First, we found that splenic NK cells from cKO mice and WT mice had similar expression of the IL-15 receptors CD122 (IL-15Rβ) and CD132 (IL-15Rγ_c_) (Fig. 6a, b). We then investigated the different signaling pathways downstream of IL-15 stimulation. While METTL3-deficient NK cells had nearly unaltered levels of STAT5 phosphorylation upon IL-15 stimulation in vitro, phosphorylation of AKT, MAPK, and ERK was significantly decreased (Fig. 6c, d, Supplementary Fig. 9A and B). m^6^A methylation in T cells inhibits expression of SOCS3 (a negative regulator of STAT5)^36,52^. Consistent with unchanged phosphorylation of STAT5, METTL3-deficient NK cells did not show altered expression of SOCS3 (Fig. 6e), suggesting that m^6^A methylation regulated NK cells in a mechanism different from that observed in T cells. AKT phosphorylation leads to mTOR activation, which facilitates NK cell metabolism^17,18^. Indeed, impaired activation of AKT was accompanied with decreased phosphorylation of mTOR in METTL3-deficient NK cells (Fig. 6f). Consequently, METTL3-deficient NK cells showed lower maximal respiration and weaker spare respiratory capacity than that of NK cells in WT mice upon IL-15 stimulation (Fig. 6g, h). Taken together, these results indicate that METTL3 is required for sufficient engagement of AKT-mTOR and MAPK-ERK signaling pathways downstream of IL-15R.

### *Ptpn11* is m^6^A modified and regulates IL-15-induced signaling

To gain insight into the mechanisms underlying the effect of METTL3-mediated m^6^A modification on the IL-15-induced signaling, we performed methylated RNA immunoprecipitation sequencing (MeRIP-seq). Consistent with the previous studies^27,53,54^, m^6^A methylation sites were located primarily in the 3′ untranslated regions (UTRs), coding sequences (CDSs) region, and near stop codons (Supplementary Fig. 10A, B), and m^6^A peaks in NK cells were significantly enriched in DRACH (D = A/G/U; R = A/G; H = A/C/U) motifs (Supplementary Fig. 10C). Further analysis revealed that m^6^A-modified targets included the genes involved in PI3K-AKT and mTOR signaling pathways (Supplementary Fig. 10D). Meanwhile, we performed METTL3-RIP-seq and found similarities in distribution and motifs of METTL3 peaks with those of m^6^A peaks (Supplementary Fig. 10E–G). Since RNA-seq analysis revealed an unchanged expression level of transcripts related to the IL-15-signaling pathway (Supplementary Fig. 10H), we conducted a proteomic analysis of NK cells from WT and cKO mice. We observed 256 downregulated proteins and 262 upregulated proteins in METTL3-deficient NK cells, with a greater than 2-fold change, compared with METTL3-sufficient NK cells (Supplementary Fig. 10I, J).

**Fig. 7:**
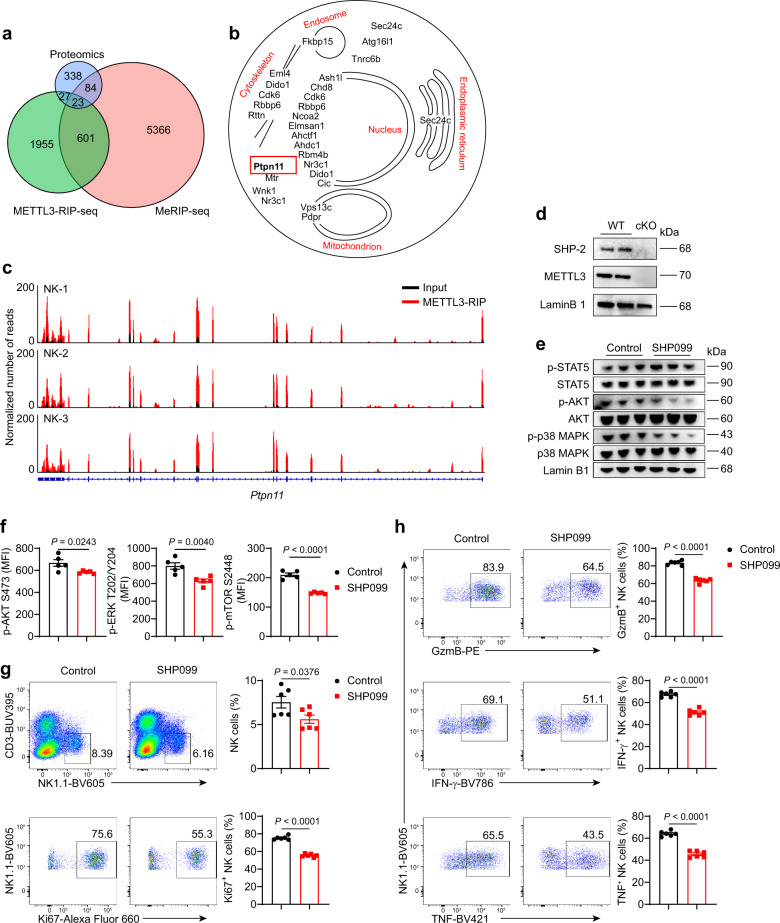
METTL3-mediated m^6^A modification promotes SHP-2 protein expression. **a** Venn diagram showing the genes or proteins modified by m^6^A, bound by METTL3, and affected by METTL3 deficiency. **b** Cellular locations of the 23 proteins in (**a**) using information obtained from Uniprot. **c** Gene track shows *Ptpn11* locus as a histogram of normalized reads (*y*-axis) plotted by genome position (*x*-axis). **d** Immunoblotting of SHP-2 and METTL3 in purified splenic NK cells from WT and cKO mice. **e** Immunoblotting of indicated proteins in purified splenic NK cells after stimulated with rmIL15 (50 ng/mL) in the presence or absence of SHP099 (4 μM) for 1 h. **f**   The splenocytes were cultured with rmIL-15 (50 ng/mL) in vitro for 3 days, followed by SHP099 (4 μM) treatment for 1 h, and then collected for flow-cytometric analyses of the indicated molecule expression in NK cells (*n* = 5/group). **g**, **h** Splenocytes from WT mice were stimulated with rmIL-15 (50 ng/mL) in the absence or presence of SHP099 (4 μM) for 3 days (*n* = 6/group). **g** Representative plots (left) showing the expression of CD3 and NK1.1 in cultured splenocytes or Ki67 expression in splenic NK cells; statistical graphs (right) showing the percentages of NK cells or Ki67^+^ NK cells. (**h**) Representative plots (left) and percentages (right) for the expression of GzmB, IFN-γ, TNF in splenic NK cells 4 hours after being stimulated with PMA and ionomycin. Each symbol represents an individual well of a cell culture plate (**f**–**h**). Data are the mean ± SEM (ns, not significant; unpaired two-tailed *t* test). Source data are provided as a Source Data file. Data represent one (**f**) or at least two independent experiments (**d**, **e**, **g**, **h**).

Conjoint analysis of MeRIP-seq, METTL3-RIP-seq, and proteome data showed that 23 genes were commonly targeted by both m^6^A and METTL3, and meanwhile, their protein products were differentially expressed between METTL3-deficient NK cells and METTL3-sufficient NK cells (Fig. 7a, b). Among them, SHP-2 encoded by *Ptpn11* has been reported to function as a critical mediator for IL-15-induced ERK activation^55^. In accordance with the proteome data, immunoblot analysis revealed decreased SHP-2 expression in METTL3-deficient NK cells (Fig. 7d). Furthermore, treatment with the SHP-2 inhibitor SHP099 significantly inhibited the phosphorylation of AKT, mTOR, ERK, and MAPK, but not the phosphorylation of STAT5 (Fig. 7e, f), and NK cell percentages and proliferation were also decreased (Fig. 7g). With a high dose of IL-15 stimulation, SHP099-treated NK cells exhibited impaired expression of GzmB, IFN-γ, and TNF (Fig. 7h). Thus, SHP-2 inhibition in WT NK cells nearly phenocopied the defects observed in METTL3-deficient NK cells. Considering decreased SHP-2 expression in METTL3-deficient NK cells at protein levels, METTL3 regulates NK cell responsiveness to IL-15 presumably through promoting SHP-2 expression.

## Discussion

m^6^A is the most common modification in mRNA. Here, we unveil a critical role of METTL3-mediated m^6^A methylation in the homeostasis and antitumor immunity of NK cells. The TME downregulated METTL3 protein expression in NK cells via TGF-β and decreased METTL3 protein expression was accompanied by weaker effector functions and reduced m^6^A levels of NK cells. Moreover, METTL3 deficiency caused a reduction of NK cells and limited terminal maturation of peripheral NK cells. Moreover, by using several tumor models, we found that tumor-bearing cKO mice displayed more aggressive tumor progression, extensive metastases, and shortened survival compared with those in WT controls. Such consequences resulted from the reduced infiltration and impaired effector functions of METTL3-deficient NK cells in a tumor *niche*. Further results demonstrated that METTL3-mediated m^6^A methylation guaranteed sufficient engagement of AKT-mTOR and MAPK-ERK signaling pathways in response to IL-15 stimulation, presumably through promoting SHP-2 expression. These findings uncover the role of m^6^A methylation in facilitating the homeostasis and tumor immunosurveillance of NK cells.

The core components of the m^6^A methyltransferase complex contain METTL3, METTL14, and WTAP. Among them, only METTL3 is the catalytically active subunit^22^. We found a positive correlation between mRNA expression of *METTL3* and NK cell effector function-related gene module in different human tumors. Further analysis revealed that TGF-β, a cytokine enriched within the TME, induced downregulation of METTL3 protein expression, while the m^6^A “reader” YTHDC1, YTHDC2, YTHDF2, and “eraser” FTO, ALKBH5 remained unchanged. Decreased METTL3 protein expression induced by TGF-β treatment or using shRNA led to reduced m^6^A levels in NK cells. These findings highlight the potential role of METTL3 in regulating NK cell functionality and m^6^A methylation. As such, we generated cKO mice with specific deletion of *Mettl3* mRNA in NKp46^+^ cells to investigate the effects of METTL3-mediated m^6^A methylation on NK cell homeostasis and anti-tumor activity. However, we did not exclude regulatory functions of other molecules for m^6^A modification in NK cells, which still deserve further investigation.

Previous studies show elevated METTL3 expression in gastric cancer tissues and acute myeloid leukemia (AML) cells, which promotes tumor cell proliferation and thereby facilitates cancer progression^28,47,56^. However, unlike the expression pattern in tumor cells, we found that tumor cells downregulated METTL3 protein expression in NK cells in a TGF-β-dependent manner, which could further impair NK cell functionality. For AML cells, METTL3-mediated m^6^A methylation promotes the translation of *c-MYC*, *BCL2*, and *PTEN*, leading to the decreased level of phosphorylated AKT^28^. In contrast, METTL3 is required for sufficient phosphorylation of AKT of NK cells in response to IL-15. These findings suggest that, with tumor progression, METTL3 expression pattern and intrinsic signaling pathway might be different for tumor cells and immune cells. Effects of METTL3 inhibition on anti-tumor immunity should be taken into consideration when evaluating METTL3 as a therapeutic target for cancer therapy.

At a steady-state, METTL3 deficiency resulted in a reduction of NK cell numbers in multi-organs, whereas the apoptosis, proliferation, and cell cycle of NK cells were barely affected. Upon tumor challenge or IL-15 treatment, NK cell reduction was more profound in cKO mice, and IL-15-driven proliferation, survival, and activation were impaired in METTL3-deficient NK cells. Regarding IL-15-induced downstream signaling pathways, we observed that METTL3 deficiency did not significantly impact the JAK-STAT5 engagement, whereas the AKT-mTOR, and MAPK-ERK signaling pathways were strongly affected. It has been reported that phosphorylation of AKT or mTOR required a high dose of IL-15, whereas a low concentration of IL-15 was sufficient to activate STAT5^49^. The enhanced changes between METTL3-deficient and METTL3-sufficient NK cells under high levels of IL-15 might reflect the differential contribution of IL-15R downstream signaling pathways under different levels of IL-15 stimuli. The JAK-STAT5 signaling pathway may be sufficient for the survival of NK cells at a steady-state with a low concentration of IL-15. However, in response to high levels of IL-15, the PI3K-AKT signaling pathway is indispensable for the proliferation and effector functions of NK cells, thereby sustaining the antitumor immunosurveillance of NK cells.

The importance of SHP-2 in mediating IL-15-induced ERK activation in NK cells has been defined, and its function of positive regulation of MAPK has also been well-characterized^55^. In our study, we found that *Ptpn11* is directly targeted by METTL3-mediated m^6^A modification via MeRIP-seq and METTL3-RIP-seq analysis. The proteomic analysis further revealed reduced protein expression of SHP-2 in METTL3-deficient NK cells, but its transcriptional expression level was unaltered. Studies have demonstrated that, in the cytoplasm, m^6^A-modified mRNAs bind to specific “reader” proteins that promote mRNA translation^20,22,23,57^. Furthermore, METTL3 could promote translation of target mRNAs via recruiting translation initiation factors^58^, and m^6^A residues within the 5′ UTR also promote translation in a cap-independent manner^59^. Considering METTL3 and m^6^A methylation can affect protein expression in different ways, the precise mechanism of translational regulation of SHP-2 by METTL3 still needs further investigation. Additionally, controlling mRNA degradation is a major mechanism of m^6^A biology. m^6^A methylation could indirectly regulate the expression of certain molecules by degrading the mRNA of their co-regulators^60^. Whether the mRNA levels of SHP-2 suppressors are degraded by m^6^A methylation, thereby affecting SHP-2 protein expression, is also unknown.

Altogether, our study reports the importance of METTL3-mediated m^6^A methylation in regulating homeostasis and anti-tumor immunity of NK cells through retention of intact IL-15-dependent signaling pathways. These findings provide insights into NK cell-based or METTL3-related therapeutic strategies for different cancer types.

## Methods

### Mice

C57BL/6 (B6) mice were purchased from the Beijing Vital River Company (Beijing, China). *Mettl3*^fl/fl^ mice were described before^36^. *Ncr1*^Cre/+^ mice were obtained from Zhongjun Dong (Tsinghua University, Beijing, China). *Ncr1*^Cre/+^-*Mettl3*^fl/fl^ mice were generated by crossing *Ncr1*^Cre/+^ mice with *Mettl3*^fl/fl^ mice. All mice had a B6 background and were maintained and treated in a specific pathogen-free facility in accordance with the ethical guidelines for experimental animals approved by the Institutional Animal Use and Care Committee at the University of Science and Technology of China. Mice aged between 6 and 10 weeks were used. Animals were maintained at 21 ± 1 °C, in 40–70% humidity, and with a 12 h light/dark cycle (from 8 a.m. to 8 p.m.).

### Peripheral blood from healthy humans

Fresh peripheral blood mononuclear cells (PBMCs) were obtained from the First Affiliated Hospital of Anhui Medical University (Hefei, China). All specimens were obtained and collected with informed consent obtained from the participants by using a protocol approved by the Ethics Review Board of the University of Science and Technology of China.

### Cell lines and plasmids

The B16/F10, 293T, and YAC-1 cell lines were purchased from the cell bank of the Chinese Academy of Sciences (Shanghai, China). The MC38 cell line was gifted by Yangxin Fu (Southwestern Medical Center, University of Texas, Houston, TX, USA). RMA cell line was gifted by Shuren Zhang (Cancer Hospital and Institute, Chinese Academy of Medical Sciences, Beijing, China). RMA-S cell line was gifted by Zhongjun Dong. NK92 cell line was purchased from the American Type Culture Collection (CRL-2407, ATCC, Manassas, VA, USA). All cell lines were tested negative for mycoplasma contamination. YAC-1, 293T, RMA, and RMA-S cell lines were cultured in RPMI 1640 medium (Hyclone, South Logan, UT, USA) and supplemented with 10% fetal bovine serum (FBS) (Gibco, Carlsbad, CA, USA) and 100 U/mL Penicillin and 0.1 mg/mL Streptomycin solution (Beyotime, Shanghai, China). B16/F10 and MC38 cell lines were cultured in DMEM medium (Hyclone) and supplemented with 10% FBS and 1% Penicillin-Streptomycin solution. NK92 cell line was cultured in αMEM (Gibco) and supplemented with 0.2 mM inositol (Sigma–Aldrich, Saint Louis, MO, USA), 0.1 mM β-mercaptoethanol (Sigma-Aldrich), 0.02 mM folic acid (Sigma-Aldrich), 100-200 U/mL recombinant IL-2 (Genscript, Nanjing, Jiangsu, China), 1% Penicillin–Streptomycin solution, 10% horse serum (Gibco) and 10% FBS. The pLIVE-IL-15 plasmid was generated as described previously^51^, and the pLIVE plasmid was used as a control. Hydrodynamic injection of pLIVE-IL-15 and pLIVE plasmids into mice was undertaken as reported previously^61^.

### Tumor models

To induce the development of liver metastases, mice were intrasplenically (i.s.) inoculated with 2 × 10^5^ MC38 or B16/F10 cells in 50 μL of PBS, followed by splenectomy 3 min after injection; PBS-injected mice were used as controls. For the induction of pulmonary metastases, mice were intravenously (i.v.) inoculated with 2 × 10^5^, 2.5 × 10^5^, or 5 × 10^5^ MC38 or B16/F10 cells in 200 μL of PBS. Mice were sacrificed at indicated time points after the injection of tumor cells. For the liver or pulmonary metastasis model, all mice died or were humanely euthanized within 10 weeks after tumor inoculation. For the subcutaneous tumor model, mice were subcutaneously (s.c.) inoculated with 2 × 10^5^ MC38 cells. Subcutaneous tumors were measured by caliper for every three days, and the volume was calculated as 0.5 × length × width × width. Mice were euthanized when tumors grew to larger than 1000 mm^3^.

### Transient transfection and lentivirus infection

To generate lentivirus to infect NK92 cells, 293T cells were co-transfected with pSLenti-U6-shRNA-CMV-EGFP-F2A-Puro-WPRE expressing *METTL3*-specific shRNA, or with corresponding control plasmids with the packaging constructs pLP/VSVG and pLP2. These three plasmids were purchased from Obio Technology Corp., Ltd. (Shanghai, China). For transduction, lentivirus was added to NK92 cells in the presence of 8 μg/mL polybrene. Then, the samples were centrifuged for 60 min at 32 °C and 1800*g* and incubated overnight at 37 °C before replacing the medium with a regular growth medium. shRNA-NK92 cells were purified by FACS with FACSAria Fusion (BD Biosciences, San Diego, CA, USA), the efficiency of knockdown was verified by flow cytometry (BD Biosciences). The sequence of shRNA for human *METTL3* was: 5′-GCAAGAATTCTGTGACTAT-3′. The sequence of shRNA for control was: 5′-CCTAAGGTTAAGTCGCCCTCG-3′.

### Cell isolation

For liver mononuclear cells (MNCs) isolation, the liver was harvested, and tissue passed through a 200-gauge mesh, which was resuspended in 40% Percoll (GE Healthcare, Chicago, IL, USA), and overlaid carefully onto 70% Percoll. After centrifugation, liver MNCs were collected from the interphase. Splenocytes were homogenized gently and erythrocytes lysed. For lung MNCs, lungs were excised, minced, and digested for 60 min at 37 °C with RPMI 1640 medium (Hyclone) containing 1 mg/mL collagenase I (Sigma-Aldrich) and 5% fetal calf serum (Gibco, Carlsbad, CA, USA). Subsequently, the digestion products were resuspended in 40% Percoll and overlaid carefully onto 70% Percoll. After centrifugation, lung MNCs were collected from the interphase.

### Depletion of NK cells

For depletion of NK cells, mice were intraperitoneal injection of 200 μg anti-NK1.1 mAb (PK136 and purified from cell supernatants in-house) or 50 μL of rabbit anti-mouse ASGM1 (Wako Pure Chemical Industries. Osaka, Japan) Ab 24 h before tumor-cell inoculation, followed by mAb injection weekly. Totally, 200 μg Rat IgG mAb or 200 μL of PBS was injected respectively as control.

### Purification of NK cells

Splenocytes were harvested from *Mettl3*^fl/fl^-*Ncr1*^Cre/+^ mice or *Mettl3*^fl/fl^ mice, and incubated with anti-NKp46 mAb (Biolegend, San Diego, CA, USA) at 4 °C, followed by labeling of anti-PE MicroBeads (Miltenyi Biotec, Bergisch Gladbach, Germany). Then NK cells were sorted with a magnetic-activated cell separation system by using LS Columns (Miltenyi Biotec). When the purity of NK cells was less than 90%, NK cells would be further sorted by using BD FACSAriaII or FACSAria Fusion (BD Biosciences). The purity of the sorted cell populations was greater than 95%.

### Antibodies and flow cytometry

The detailed information about flow antibodies used in this study was provided in Supplementary Table 1.

For staining of cell surface molecules, cells were pre-incubated with rat serum to block Fc receptors and stained with indicated antibodies for 30 min at 4 °C.

For staining of intracellular Ki67, cytokines or granules, cells were stimulated for 4 h with 30 ng/mL PMA (Sigma-Aldrich) and 1 μM ionomycin (Sigma-Aldrich) in the presence of 2.5 μg/mL monensin (eBioscience, San Diego, CA, USA). After stimulation, cells were stained for surface markers, fixed, and permeabilized with FoxP3 fixation buffer (Thermo Fisher Scientific) according to manufacturer instructions. Fixed cells were stained with indicated antibodies. For CD107a staining, the CD107a mAb was incubated with the cells at the beginning of PMA/ionomycin stimulation. For staining of transcription factors, METTL3 and Bcl2, cells were fixed, permeabilized with FoxP3 fixation buffer, following staining of cell surface molecules, and then stained with indicated antibodies.

For intracellular phospho-staining, cells were firstly surface stained, followed by stimulated with rmIL-15 (50 ng/mL) for 1 h. After stimulation, cells were fixed with BD Phosflow Fix Buffer I (BD Biosciences) and permeabilized with BD Phosflow Perm Buffer III (BD Biosciences) according to manufacturer instructions. Fixed cells were stained with indicated phospho-antibodies.

Sample events were collected on BD flow cytometers (BD LSR II, LSR Fortessa, and LSRfortessa X20; BD Biosciences) and were analyzed using FlowJo (Tree Star, Ashland, OR, USA).

### m^6^A RNA methylation quantification

TGF-β-treated mouse NK cells and the shRNA-NK92 cells were resuspended in TRIzol (Thermo) for total mRNA extraction by using the Direct-zol RNA Miniprep kit (Zymo Research, CA, USA, Cat # R2052) according to manufacturer instructions. mRNA concentration was detected with NanoDrop one (Thermo). The process of m^6^A methylation quantification was performed by EpiQuik m6A RNA Methylation Quantification Kit (Colorimetric) (Epigentek, NY, USA, Cat # P-9005-96) according to manufacturer instructions.

### Labeling of RMA cells and RMA-S cells and in vivo cytotoxicity

RMA cells or RMA-S cells were labeled, respectively, with CellTrace CFSE or Violet (CTV) Cell Proliferation Kit according to manufacturer (Thermo Fisher Scientific, Cat # C34554 for CFSE, Cat # C34557A for CTV) instructions. Briefly, RMA cells or RMA-S cells were resuspended with RPMI 1640 medium (10^6^/mL), and mixed with an identical volume of staining solution of CFSE or CTV (final concentration = 5 μM). Cells were incubated for 20 min at 37 °C, and then RPMI 1640 medium was added, followed by incubation at 37 °C for 5 min and centrifugation. The cell pellet was resuspended with PBS. CFSE-labeled RMA (5 × 10^6^) and CTV-labeled RMA-S cells (1.5 × 10^7^) were mixed well and transferred (i.v.) into WT mice or cKO mice. Sixteen hours later, recipient mice were harvested for flow cytometry.

### Labeling of YAC-1 and in vitro cytotoxicity

YAC-1 cells were labeled with CTV (Thermo Fisher Scientific) as described above. Splenocytes were stimulated with recombinant mouse (rm) IL-12 (20 ng/mL, Peprotech), rmIL-15 (20 ng/mL, Peprotech) and rmIL-18 (10 ng/mL, R&D, Minneapolis, MN, USA) for 18 h. Then, the stimulated splenocytes (5 × 10^6^) were cocultured with CTV-labeled YAC-1 cells (5 × 10^4^) for 4 h, followed by an analysis of the BD flow cytometer.

### Staining with hematoxylin and eosin

Tissues of the liver and lung were fixed in 4% paraformaldehyde and embedded in paraffin. Tissue sections of 5 μm thickness were cut and stained with hematoxylin and eosin, which was carried out by Servicebio (Wuhan, China).

### Cell-cycle detection

The general procedure has followed the instructions of the manufacturer (BD Pharmingen BrdU Flow kits, Cat # 51-2354AK). Briefly, splenocytes from WT or cKO mice were treated with BrdU (1 mM) for 24 h, followed by cell-surface staining and fixation and permeabilization. The splenocytes were then treated with DNase and stained with anti-BrdU Abs. Finally, 7-AAD was added, and the cells were collected for flow cytometry.

### Immunofluorescence

For staining of NK cells and IL-15, paraffin sections of the lung- and liver tissue were de-waxed following fixation and embedding, rehydrated, and subjected to heat-induced epitope retrieval. Then, sections were incubated with polyclonal goat anti-mouse NCR1 (R&D) or polyclonal rabbit anti-mouse IL-15 (Thermo) at 4 °C overnight, followed by incubation with Alexa Fluor 647 Donkey anti-Goat IgG or Alexa Fluor 647 Donkey anti-Rabbit IgG (Thermo) respectively at 37 °C for 1 h. Thereafter, sections were stained with DAPI (Thermo) for 5 min and scanned by an LSM880 confocal laser scanning microscope (Carl Zeiss, Gottingen, Germany), followed by analyses by using ZEN 2012 and ZEN blue 3.1 (Carl Zeiss).

### ELISA

Purified NK cells were resuspended with RPMI 1640 complete medium and plated on a 96-well plate (5 × 10^5^ per 200 μL per well). NK cells were treated with rmIL-15 (50 ng/mL; Peprotech,) for 9 days. The concentration of IFN-γ and GzmB in the NK-cell culture supernatant was measured by ELISA kits (Multisciences, Hangzhou, China, Cat # EK280/3-96 for IFN-γ, Cat # EK2173-96 for Gzm B) according to manufacturer instructions.

### RT-qPCR

 For cytokine detection in the TME, total mRNA was isolated from liver tissue using TRIzol Reagent (Invitrogen, Carlsbad, CA, USA). RT-qPCR was done using TB Green Premix Ex Taq (TaKaRa Biotechnology, Shiga, Japan) with Light Cycler 96 (Roche, Basel, Switzerland) according to manufacturer instructions. For analyses, the expression of target genes was normalized to that of the housekeeping gene *β-actin*. cDNA fragments were amplified using the gene-specific primers provided in Supplementary Table 2.

### Immunoblotting

Fresh purified NK cells were lysed in M-PER Mammalian Protein Extraction Reagent (Thermo). Then, samples were run on precast 4–12% Bis–Tris protein gels (Genscript). Separated proteins were transferred to polyvinylidene fluoride (PVDF) membranes and blocked with 5% *w/v* skimmed milk at room temperature for 1 h. Then, PVDF membranes were incubated with primary antibodies in 5% w/v skimmed milk in Tris-buffered saline containing 0.1% Tween-20 at 4 °C overnight, then incubated with Anti-rabbit IgG, HRP-linked Ab (Cell Signaling Technology, Danvers, MA, USA) for 1 h at room temperature. To detect several proteins on the same PVDF membrane, membranes were processed with western stripping buffer (Beyotime) and reincubated with primary Abs. Chemiluminescence autoradiography was used to detect protein bands. The primary Abs for METTL3, WTAP, YTHDC2, YTHDC1, YTHDF1, ALKBH5, FTO, SHP2, AKT, p-AKT S473, STAT5, p-STAT5 Y694, Lamin B1, mTOR, p-mTOR Ser2448, p38 MAPK, p-p38 MAPK Thr180/Tyr182, and β-actin were purchased from Cell Signaling Technology; primary Ab SOCS3 was purchased from Abcam. The Anti-rabbit IgG, HRP-linker antibody was purchased from Cell Signaling Technology. The dilution of all antibodies used for immunoblotting was 1:1000.

### Metabolic assays

Measurement of the oxygen consumption rate (OCR) was conducted by using Agilent Seahorse XF Cell Mito Stress Test Kit (Agilent, Cedar Creek, Texas, USA, Cat # 103015-100) according to manufacturer instructions. Briefly, splenic NK cells (4 × 10^5^ cells per well) were plated on Cell and Tissue Adhesive (Corning, Corning, NY, USA)-pretreated Seahorse plates in XF media (25 mM glucose, 2 mM glutamine, 1 mM pyruvate) and evaluated using an XF-96 Extracellular Flux Analyzer (Agilent Technologies, Santa Clara, CA, USA). The basal OCR was measured for 30 minutes. Cells were treated with oligomycin (2 mM), Carbonyl cyanide-4-(trifluoromethoxy) phenylhydrazone (FCCP, 1.5 mM), and 1 mM-each of rotenone and antimycin A to measure maximum respiration and excess respiratory capacity. All reagents were purchased from Agilent Technologies.

### Cell culture and cytokine treatment

To study the responsiveness of NK cells to IL-15, splenocytes were resuspended with RPMI 1640 medium and plated on 24-well plates. Then splenocytes were treated with rmIL-15 (Peprotech, 100 ng/mL) for 1, 2, 3, 5, 7, or 9 days, followed by flow cytometry. To investigate the influence of cytokines on METTL3 expression in NK cells, we treated splenocytes with rmIL-17F (25 ng/mL, Peprotech), rmIL-27 (25 ng/mL, Peprotech), rmIL-10 (25 ng/mL, Peprotech), rmIL-15 (25 ng/mL, Peprotech), rmTGF-β (25 ng/mL, R&D) and rmIL-18 (25 ng/mL, R&D) for 3 days. To study the effect of MC38 cells on METTL3 expression in NK cells, purified NK cells were cocultured with MC38 cells under the stimulation of rmIL-15 (10 ng/mL) with the presence or absence of SIS3 (20 μM, APExBIO, Houston, TX, USA) or GW788388 (20 μM, APExBIO) for 2 days. To observe the effects of SHP-2 on the NK cells, splenocytes were treated with rmIL-15 (50 ng/mL, Peprotech) with or without SHP-2 inhibitor SHP099 (4 μM, Selleck, Houston, TX, USA) for 3 days.

### mRNA-seq and analyses

Splenic NK cells from WT or cKO mice were purified via FACS using BD FACSAriaII (BD Biosciences). Total mRNA was extracted using the Direct-zol RNA Miniprep kit (Zymo Research). RNA-seq library preparation and high-throughput sequencing were performed by Novogene (Beijing, China) using Illumina Hiseq PE150. RNA-sequencing reads were aligned to the mouse reference GRCm38 genome with Hisat2 (Version 2.1.0). The number of counts was summarized at the gene level with featureCounts (Version 1.5.0). The following jobs were conducted in the R statistical environment (Version 4.0.5). Differential gene expression analysis was performed with DEseq2 (Version 1.28.1). Genes with *P* < 0.05 and log_2_FC (fold change) > 1 or log_2_FC < −1 were considered differentially expressed. The volcano plot was drawn with R package ggplot2 (Version 3.3.3). The gene list related to the IL-15R pathway was supplied in Supplementary Table 3.

### MeRIP-seq and analyses

For MeRIP-seq of m^6^A, we amplified NK cells in vivo by injection of the pLIVE-IL-15 plasmid for 1–2 months. Splenic NK cells were sorted using BD FACSAriaII (BD Biosciences). Total mRNA was extracted using the Direct-zol RNA Miniprep kit (Zymo Research). MeRIP-sequencing was carried out by LC-BIO Biotech (Hangzhou, China). Briefly, the quality and quantity of total RNA were evaluated with RNA integrity number > 7.0. Poly (A) mRNA was purified and fragmented into oligonucleotides of length ~100 nt. Then, the cleaved RNA fragments were incubated for 2 h at 4 °C with an m^6^A-specific Ab (Synaptic Systems, Goettingen, Germany) in IP buffer (50 mM Tris-HCl, 750 mM NaCl, 0.5% Igepal CA-630) supplemented with bovine serum albumin (0.5 μg/μL). The mixture was incubated with protein-A beads and eluted with elution buffer. Eluted m^6^A-containing fragments and untreated input control fragments were converted to a final cDNA library. The paired-end 2 × 150-bp libraries were sequenced on an Illumina Novaseq 6000 platform at LC-BIO Biotech Limited following their recommended protocol. For analyses of MeRIP-seq results, FastQC (Version 0.11.5), Trimmomatic (Version 0.36), and Cutadapt (Version.1.10) were used for Data Quality Control. HISAT2 (Version.1.0) were used to map reads to the genome of Mus musculus (Version.V90). The R package exomePeak (Version.1.8) was used for Peak Calling and analysis of differential Peaks. CHIPseeker (Version.1.0) was used for Peak annotation. HOMER (Version 4.1) was used for de novo motif finding. The Kyoto encyclopedia of genes and genomes (KEGG) analysis for annotated genes were implemented by KOBAS software (Version.2.1.1) with a *P* value cutoff of 0.05 to judge statistically significant enrichment.

### METTL3-RIP-seq and analyses

For METTL3-RIP-seq, we collected NK cells 1-month post hydrodynamic injection of the pLIVE-IL-15 plasmid. Splenic NK cells of WT mice were sorted using BD the FACSAria Fusion cell sorter (BD Biosciences). NK cells were treated with cell lysis buffer. The 20% lysis sample was stored and named “input”, and 80% was used in immunoprecipitation reactions with anti-METTL3 Ab (Abcam, ab195352) and named “IP”. The RNA of input and IP was extracted and the stranded RNA sequencing library was constructed. The library products corresponding to 200–500 bps were enriched, quantified, and finally sequenced on Novaseq 6000 sequencer (Illumina) with PE150 model. The quality-controlled clean data were mapped to the reference genome of mus musculus GRCm38 using STAR software (Version 2.5.3a) with default parameters. The RSeQC (Version 2.6) was used for reads distribution analysis. The exomePeak (Version 3.8) software was used for peak calling. Peaks were annotated using bedtools (Version 2.25.0). Identified METTL3 peaks with a bed or bam format were adapted for visualization on the IGV software (http://www.igv.org/). The deepTools (Version 2.4.1) were used for peak distribution analysis. The differentially binding peaks were identified by a python script, using the fisher test. De novo sequence motifs enriched in peak regions were identified using Homer (Version 4.10).

### Assay of TCGA datasets

The gene set utilized for the effector-function signatures of NK cells included *NCR1*, *TBX21*, *EOMES*, *CD69*, *DNAM1*, *KLRK1*, *PERF*, *IFNG*, and *GZMB*. Analyses of correlation were undertaken using the GEPIA2 Internet website (http://gepia2.cancer-pku.cn/#index).

### Analyses of microarray and RNA-seq

The microarray data were obtained from liver NK cells of HCC patients or benign patients (GEO accession: GSE120123), including intratumor NK cells, tumor-edge NK cells, and two samples of normal liver NK cells. Differentially expressed genes (DEGs) were analyzed with limma package (3.48.3). The RNA-seq data (GEO accession: GSE153713) of ascites NK cells from ovary cancer patients and normal NK cells from healthy donor peripheral blood. DEGs were analyzed with edgeR package (3.30.3). The NK cell effector function-related genes were selected according to NK cell-related gene sets provided by GSEA. Heatmap plots were drawn with R package pheatmap (Version 1.0.12).

### Mass spectrometry and analyses

Purified NK cells were sorted from splenocytes of WT or cKO mice. The protein extraction and mass spectrometry analysis were conducted by Proteomics Platform of the Human Phenome Institute of Fudan University (Shanghai, China). Briefly, cell samples were lysed, then followed by an ultrasonic break. Protein concentration was determined by the BCA method. The same amount of protein was taken for disulfide bond cleavage (DTT) and reductive alkylation (IAA), followed by adding an appropriate amount of NH_4_HCO_3_ solution and trypsin to hydrolyze sulfate. After enzymolysis, an appropriate amount of acetonitrile (ACN) and formic acid (FA) mixed solution was added to the sample for extraction, and centrifuging at 60 °C to drain. After adding 0.1% FA solution to reconstitute, we performed homemade C18 desalination. ACN solution was used to activate the C18 column and equilibration the C18 column with FA solution. At last, samples were loaded and replace with the peptide was with ACN solution, centrifuging at 60 °C and draining it for mass spectrometry analysis by using EASY-Nlc1200 (HFX, Thermo). MaxQuant was used for sample analyses, and there were 2364-3372 proteins identified in 8 samples.

### Statistical information

Statistical significance was determined using Prism 8.0.2 (GraphPad, San Diego, CA, USA). Two-tailed unpaired or one-way analysis of variance (ANOVA) across multiple groups was used to determine significance. Correlations between variables were evaluated using the Spearman rank correlation test for data from TCGA and GTEx. Correlations between variables were examined using the Pearson rank correlation test for data from mice. The difference in overall survival of mice was assessed using the log-rank test. The differences in Supplementary Fig. 3G and Fig. 5e–h were tested using two-way ANOVA. *P* < 0.05 was considered significant.

### Reporting summary

Further information on research design is available in the Nature Research Reporting Summary linked to this article.

## Supplementary information

Supplementary Information

Reporting Summary

## Data Availability

The mRNA-seq data of NK cells from WT or cKO mice generated in this study have been deposited in the NCBI Gene Expression Omnibus with primary accession code GSE180053. The MeRIP-sequencing and METTL3-RIP-sequencing data generated in this study have been deposited in the NCBI GEO with primary accession codes GSE173326 and GSE173325, respectively. The mass spectrometry proteomics data have been deposited to the ProteomeXchange Consortium (http://proteomecentral.proteomexchange.org) via the iProX partner repository^62^ with the project IPX0003021000 and dataset identifier PXD025823 (https://www.iprox.cn//page/project.html?id=IPX0003021000). The microarray data used in this study (GEO accession code: GSE120123) of liver NK cells (intratumor NK cells, tumor-edge NK cells, and normal liver NK cells) from HCC patients or benign patients are publicly available and were downloaded from (https://www.ncbi.nlm.nih.gov/geo/query/acc.cgi?acc=GSE120123). The RNA-seq data used in this study (GEO accession code: GSE153713) of ascites NK cells (Healthy and Patient) from ovary cancer patients and normal NK cells from healthy donor peripheral blood are publicly available and were downloaded from (https://www.ncbi.nlm.nih.gov/geo/query/acc.cgi?acc=GSE153713). Source data are provided with this paper.

## Source data

Source Data
