# Supplementary material for: METTL3-mediated m6A RNA methylation promotes the anti-tumour immunity of natural killer cells
Source: Nat Commun. 2021 Sep 17;12:5522. doi: 10.1038/s41467-021-25803-0 (PMC8448775; doi:10.1038/s41467-021-25803-0)
Supplement: Supplementary file 1 — Supplementary Information [file 41467_2021_25803_MOESM1_ESM.pdf]

Supplementary Information

METTL3-mediated m^6A RNA methylation promotes the anti-tumour immunity of natural killer cells

Hao Song *et al.*

Supplementary Figures 1-10

Supplementary Tables 1-3

Supplementary Figure 1

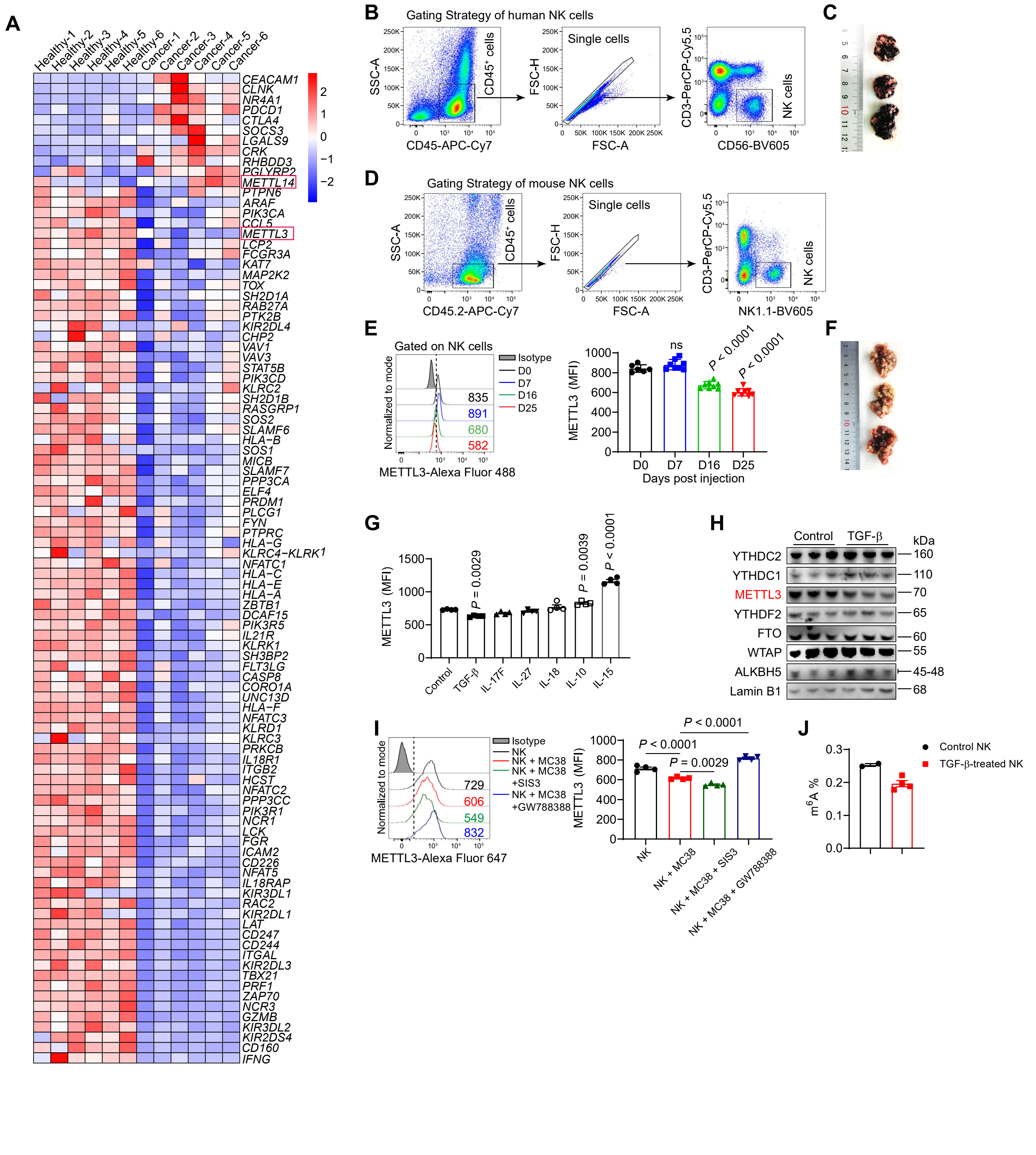

Supplementary Fig. 1

TGF-β induces decreased expression of METTL3 in NK cells in the TME.

(A) Heatmap showing the NK cell effector function-related genes in ascites NK cells from ovary cancer and NK cells from healthy donor peripheral blood. Data were collected from a public dataset (GSE153713).

(B) Gating strategy for detection of METTL3 within human NK cell populations.

(C) Imaging of lungs from tumor-bearing mice described in Fig. 1d-h.

(D) Gating strategy for detection of METTL3 within mouse NK cell populations. The same gating strategy was applied on all analyses of protein expression on NK cells, except for additional descriptions.

(E) Representative histogram (left) and MFI (right) of METTL3 expression in lung NK cells at indicated time points after intravenous injection of 5×10^5 B16/F10 (n = 6 for D0 group; n = 8 for D7, D16 and D25 groups).

(F) Imaging of livers from tumor-bearing mice described in Fig. 1i-m.

(G) MFI of METTL3 expression in NK cells following stimulation of the indicated cytokines (25 ng/mL) for 3 days (n = 4/group).

(H) Immunoblotting show the expression of m^6A-related proteins in purified NK cells treated with or without TGF-β (25 ng/mL) for 3 days.

(I) Representative histogram (left) and MFI (right) of METTL3 expression in purified NK cells after co-culture with MC38 cells with IL-15 (10 ng/mL) in the absence or presence of SIS3 (20 μM) or GW788388 (20 μM) (n = 4/group) for 2 days.

(J) Overall levels of mRNA m^6A methylation in NK cells treated with or without TGF-β (25 ng/mL) for 3 days (n = 2 for Control NK group; n = 4 for TGF-β treated NK group).

Each symbol represents an individual mouse (E), or individual well in a cell culture plate (G, I, J). Data are the mean ± SEM (ns, not significant; one-way ANOVA (E, G, I)). Source data are provided as a Source Data file. Data represent at least two independent experiments (B-J).

Supplementary Figure 2

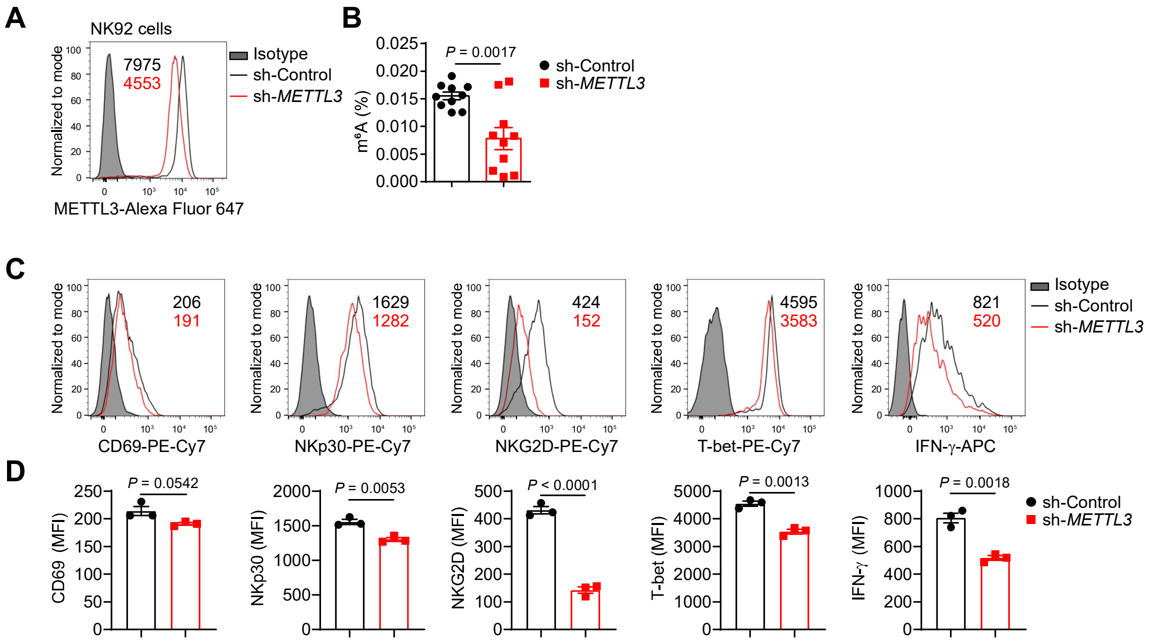

Supplementary Fig. 2

METTL3 correlates with m^{6}A levels and functions of NK92 cells.

(A-D) NK92 cells were transduced with lentiviruses expressing a scramble shRNA (sh-Control) or a shRNA targeting *METTL3* (sh-*METTL3*).

(A) Representative histogram showing the METTL3 expression in sh-Control-NK92 or sh-*METTL3*-NK92 cells.

(B) Overall levels of mRNA m^{6}A methylation in sh-Control-NK92 or sh-*METTL3*-NK92 cells (n = 10/group).

(C and D) Representative histograms (C) and MFI (D) for the expression of the indicated molecules in sh-Control-NK92 or sh-*METTL3*-NK92 cells (n = 3/group).

Each symbol represents an individual well in a cell culture plate (B, D). Data are the mean ± SEM (unpaired two-tailed *t*-test). Source data are provided as a Source Data file. Data represent at least two independent experiments (A-D).

Supplementary Figure 3

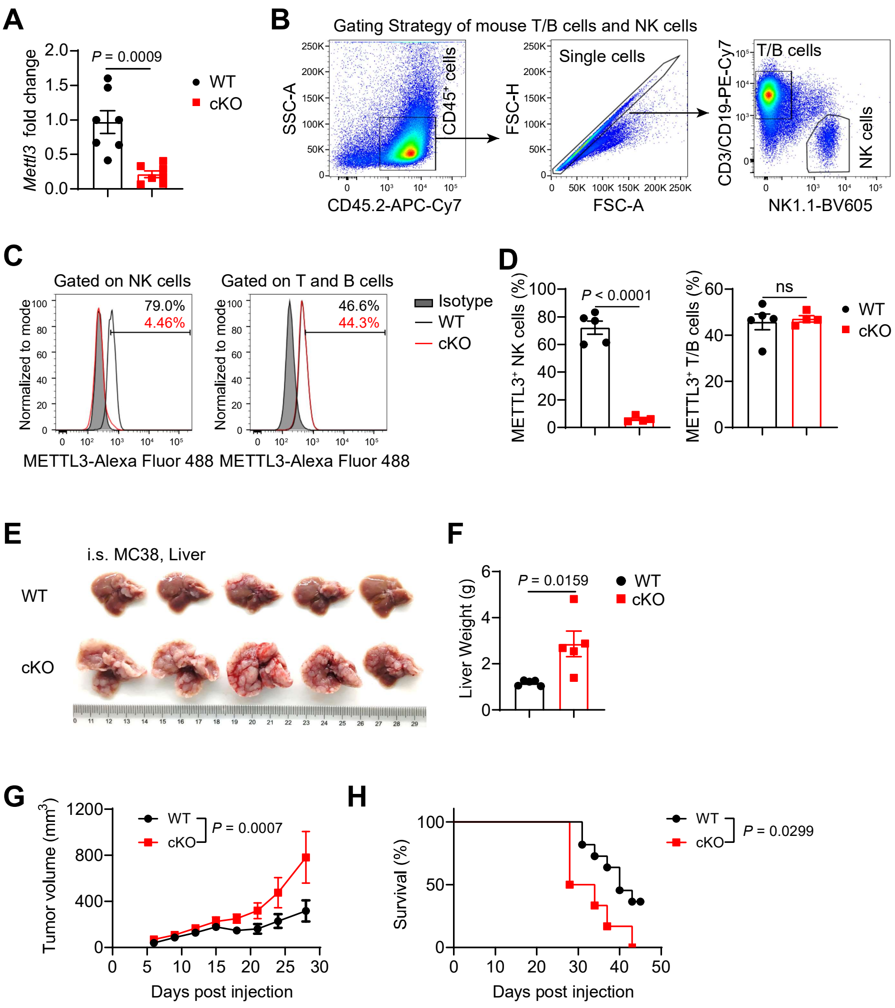

Supplementary Fig. 3

cKO mice display accelerated tumor progression.

(A) Fold change of *METTL3* expression in purified NK cells from WT (n=7) and cKO (n=7) mice, determined by RT-qPCR and normalized to *β-actin* expression.

(B) Gating strategy for detection of METTL3 within NK cell ($CD45^{+}CD3^{-}CD19^{-}NK1.1^{+}$) or T/B ($CD45^{+}NK1.1^{-}CD3^{+}/CD19^{+}$) cell populations.

(C) Representative histograms of the METTL3 in splenic NK cells or T/B cells of WT mice or cKO mice.

(D) Percentage of METTL3-positive cells among NK cells or T/B cells of WT mice (n = 5) or cKO mice (n = 4).

(E and F) Imaging (E) and weight (F) of livers from WT (n = 5) or cKO (n = 5) mice 16 days after intrasplenic injection (i.s.) of 2×10^{5} MC38 cells.

(G and H) Tumor volume (G) and survival (H) of WT (n = 11) or cKO (n = 6) mice inoculated with 2×10^{5} MC38 cells subcutaneously.

Each symbol represents an individual mouse (D, F, H) or individual well in a cell-culture plate (A). Data are the mean ± SEM (ns, not significant; unpaired two-tailed *t*-test (A, D, F) or two-tailed log-rank (Mantel–Cox) test (H) or two-way ANOVA (G)). Source data are provided as a Source Data file. Data represent one (A) at least two (C-H) independent experiments.

Supplementary Figure 4

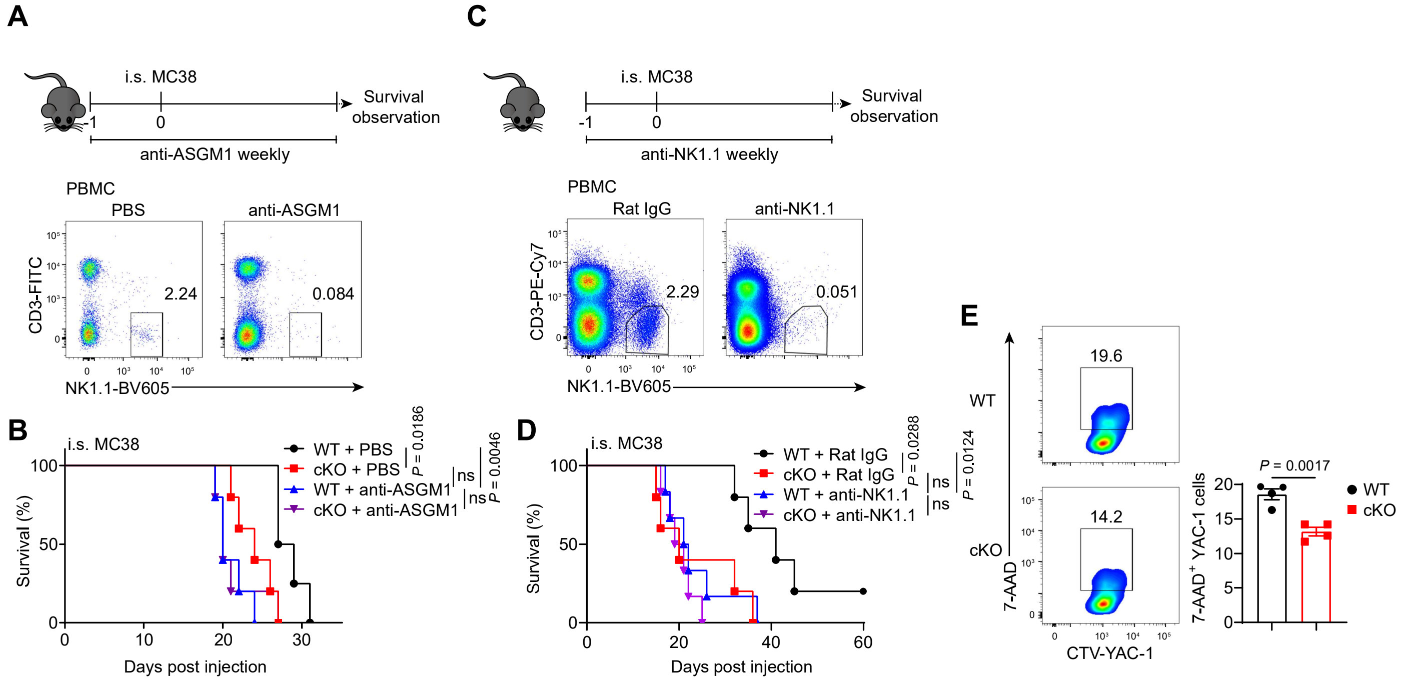

Supplementary Fig. 4

Accelerated tumor progression in cKO mice depends on NK cells.

(A) WT or cKO mice were treated with anti-ASGM1 for depletion of NK cells, followed by intrasplenic injection (i.s.) of 2×10^5 MC38 cells, and depleting efficiency was assessed by flow cytometry.

(B) Survival of WT mice (n = 4), cKO mice (n = 5), anti-ASGM1-treated WT mice (n = 5) and anti-ASGM1-treated cKO mice (n = 5) in (A).

(C) WT or cKO mice were treated with anti-NK1.1 for depletion of NK cells, followed by intrasplenic injection of 2×10^5 MC38 cells, and depleting efficiency was assessed by flow cytometry.

(D) Survival of WT mice (n = 5), cKO mice (n = 5), anti-NK1.1-treated WT mice (n = 6) and anti-NK1.1-treated cKO mice (n = 6) in (C).

(E) Splenocytes were pre-activated by cytokine stimulation and then co-cultured with CTV-labeled YAC-1 cells. Representative plots showing 7-AAD staining (left) and percentages of 7-AAD$^+$ cells (right) in CTV-labeled YAC-1 cells (n = 4/group).

Each symbol represents an individual mouse (B, D) or individual well in a cell-culture plate (E). Data are the mean ± SEM (ns, not significant; unpaired two-tailed *t*-test (E) or two tailed log-rank (Mantel–Cox) test (B, D)). Source data are provided as a Source Data file. Data represent one (B, D) or at least two independent experiments (A, C, E).

Supplementary Figure 5

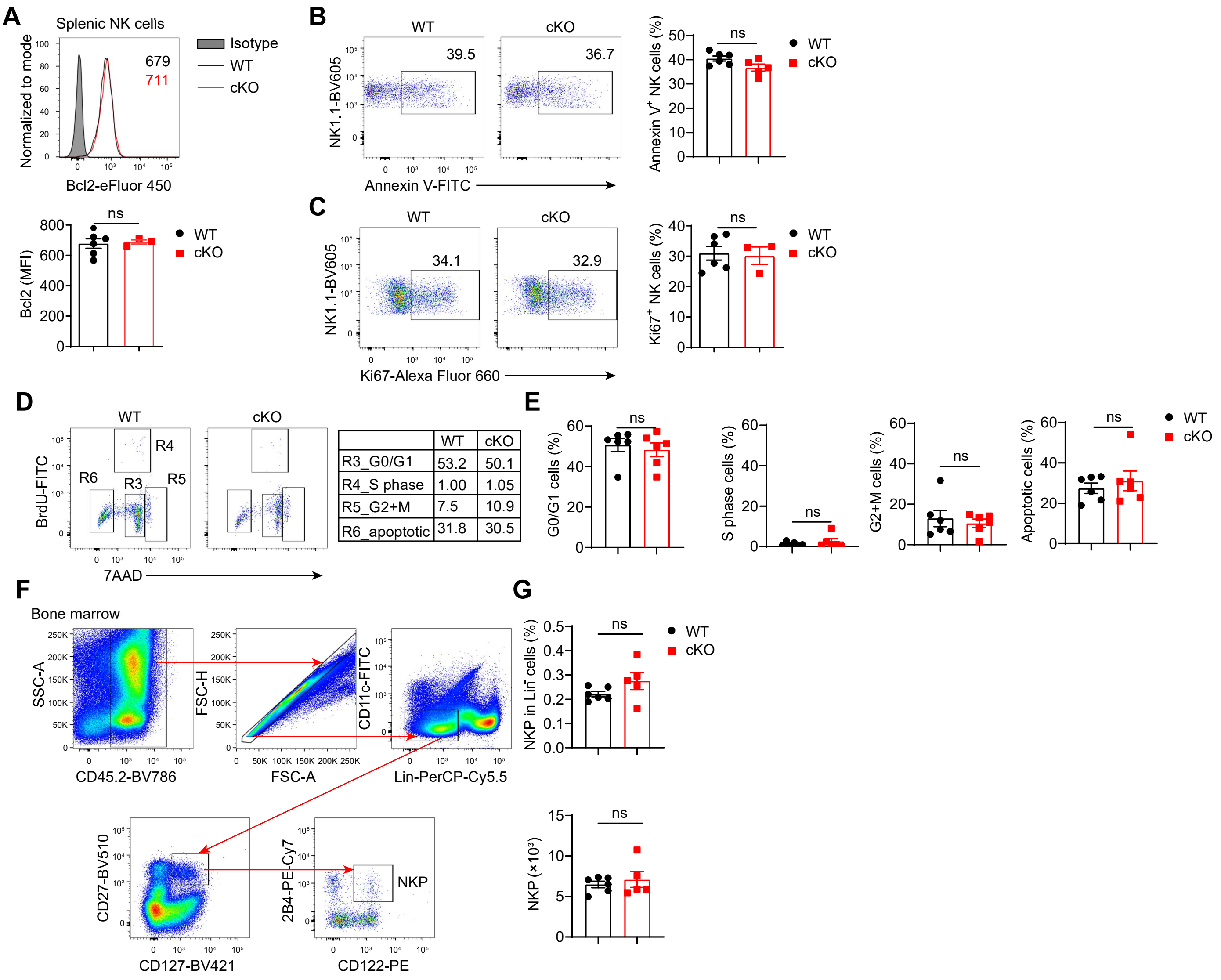

Supplementary Fig. 5

Unaltered NK cell turnover and NKP numbers in cKO mice.

(A) Representative histogram (top) and MFI (bottom) of Bcl2 expression in splenic NK cells from WT (n = 6) or cKO (n = 3) mice.

(B) Representative plots showing Annexin V staining (left) and percentage of Annexin V^+ cells (right) in NK cells from WT (n = 6) and cKO (n = 5) mice.

(C) Representative plots (left) and percentages (right) of Ki67 expression in NK cells from WT (n = 6) and cKO (n = 3) mice.

(D) Representative plots showing the expression of BrdU and 7-AAD in NK cells.

(E) Percentages of NK cells at the indicated cell-cycle stages from WT (n = 6) and cKO (n = 6) mice.

(F) Representative plots showing gating strategy of NK precursors (NKPs) in bone marrow. Lin represents antibodies including Gr1, CD11b, Ter119, CD3, CD19, and NK1.1.

(G) Percentages (top) and number (bottom) of bone marrow NKPs from WT (n = 6) or cKO (n = 5) mice.

Each symbol represents an individual mouse (A, B, C, E, G). Data are the mean ± SEM (ns, not significant; unpaired two-tailed *t*-test). Source data are provided as a Source Data file. Data represent at least two independent experiments (A-G).

Supplementary Figure 6

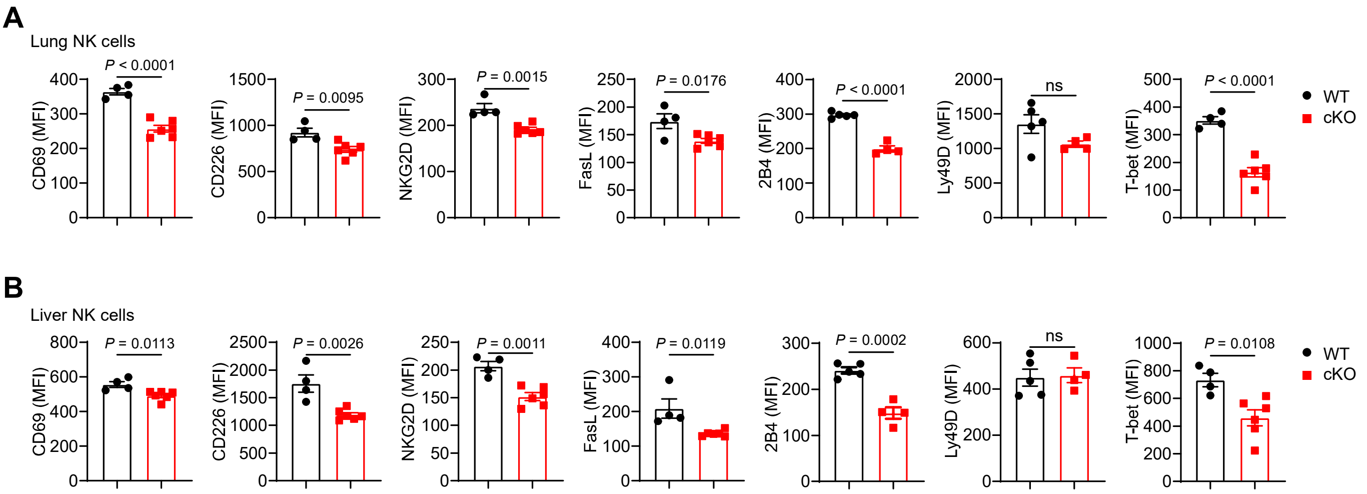

Supplementary Fig. 6

Decreased activation molecule expression on NK cells of cKO mice.

(A and B) MFI of the indicated molecule expression in lung (A) and liver (B) NK cells from WT (n = 5 for 2B4 and Ly49D, n = 4 for other molecules) or cKO mice (n = 4 for 2B4 and Ly49D, n = 6 for other molecules).

Each symbol represents an individual mouse (A, B). Data are the mean ± SEM (ns, not significant; unpaired two-tailed *t*-test). Source data are provided as a Source Data file. Data represent at least three independent experiments (A-B).

Supplementary Figure 7

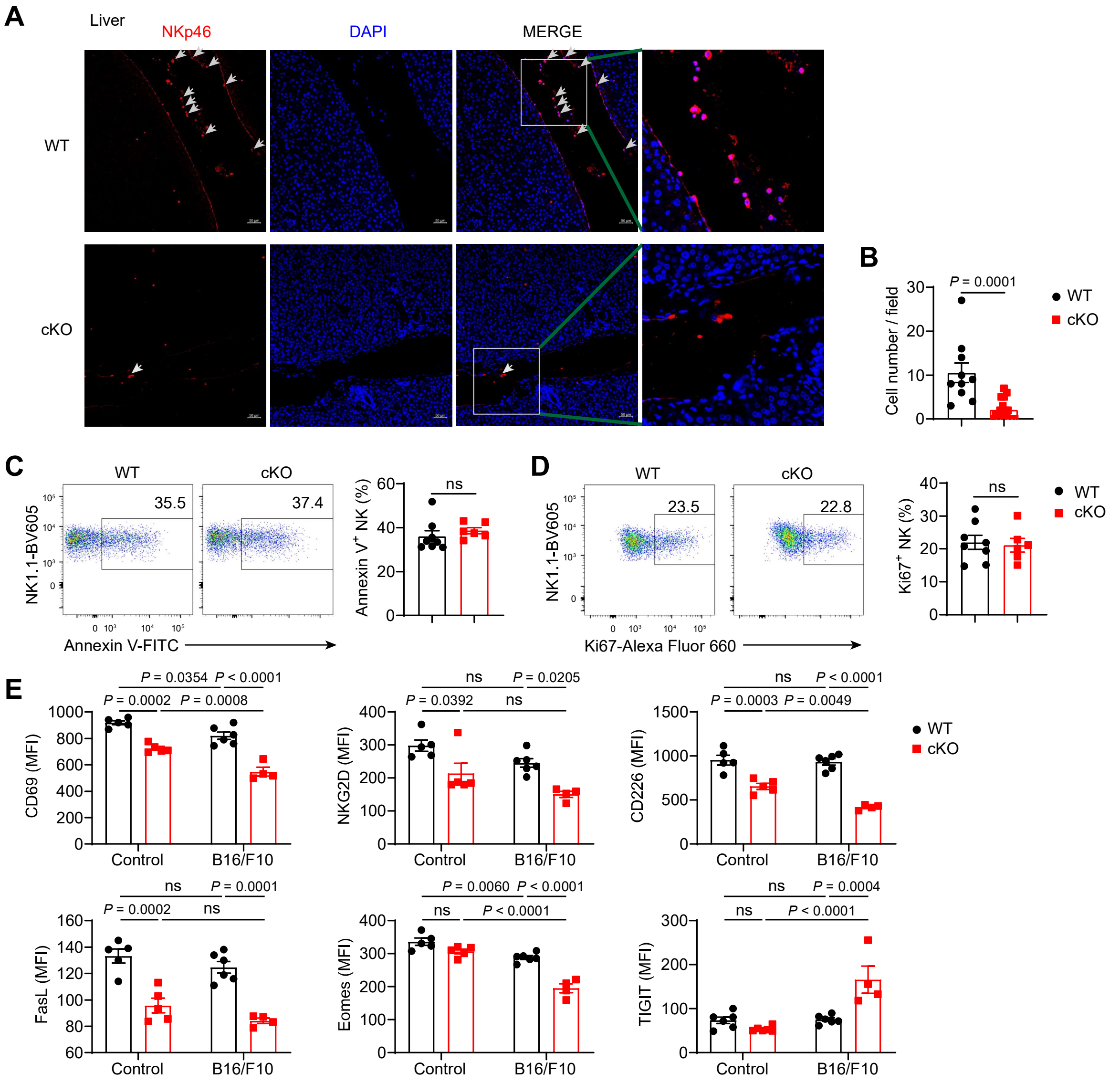

Supplementary Fig. 7

METTL3 supports NK cell infiltration and activation in the TME.

(A) Immunofluorescence staining of the liver with anti-NKp46 (red) and DAPI (blue) from WT or cKO mice 25 days after intravenous injection with 2×10^5 B16/F10 cells. Scale bar, 50 μm. The arrows indicate NK cells.

(B) NKp46-positive cell number per field (n = 10 for WT group; n = 17 for cKO group).

(C) Representative plots showing Annexin V staining (left) and percentage of Annexin V^+ cells (right) in lung NK cells from WT (n = 8) and cKO (n = 6) mice 16 days after intravenous injection with 5×10^5 B16/F10 cells.

(D) Representative plots (left) and percentages (right) of Ki67 expression in lung NK cells from WT (n = 8) and cKO (n = 6) mice as indicated in (C).

(E) MFI of the indicated molecule expression in liver NK cells from WT (n = 6 (TIGIT) or 5 (other molecules) for control group; n = 6 for B16/F10 group) or cKO mice (n = 6 (TIGIT) or 5 (other molecules) for control group; n = 4 for B16/F10 group) 23 days after intravenous injection with 2×10^5 B16/F10 cells.

Each symbol represents an individual mouse (C, D, E) or individual field of microscope (B). Data are the mean ± SEM (ns, not significant; unpaired two-tailed *t*-test (B, C, D) or one-way ANOVA (E)). Source data are provided as a Source Data file. Data represent one (A-B) or two (C-E) independent experiments.

Supplementary Figure 8

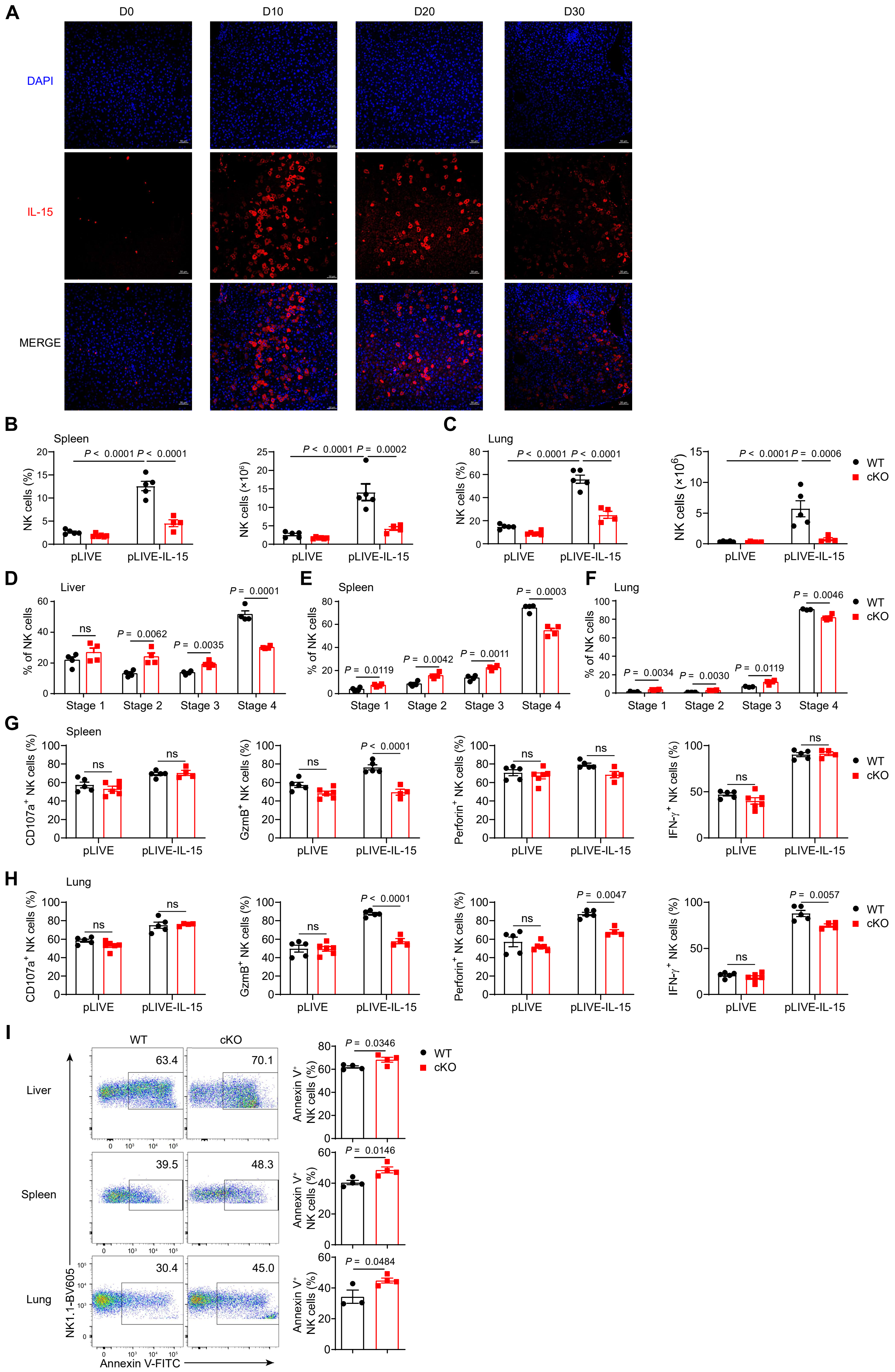

Supplementary Fig. 8

Aberrant responses of METTL3-deficient NK cells to IL-15 in vivo.

(A) Immunofluorescence staining of liver section with anti-IL-15 (red) and DAPI (blue) from WT mice after hydrodynamic injection of the pLIVE-IL-15 plasmid. Scale bar, 50 μm.

(B–I) WT mice and cKO mice were harvested one or two months after hydrodynamic injection of the pLIVE-IL-15 plasmid or pLIVE control plasmid, followed by flow cytometry. (B and C) Percentage and absolute number of spleen (B) or lung (C) NK cells from WT mice (n = 5/group) or cKO mice (n = 6 for pLIVE group; n = 4 for pLIVE-IL-15 group).

(D–F) Percentages of NK cells at different stages in the liver (D), spleen (E), and lung (F) of pLIVE-IL-15-treated WT mice (n = 3 for lung and n = 4 for liver and spleen) or cKO mice (n = 4) (Stage 1: $CD27^{-}CD11b^{-}$; Stage 2: $CD27^{+}CD11b^{-}$; Stage 3: $CD27^{+}CD11b^{+}$; Stage 4: $CD27^{-}CD11b^{+}$).

(G-H) Percentages of CD107a-, GzmB-, perforin-, or IFN-γ-positive cells among splenic (G) or pulmonary (H) NK cells 4 hours after stimulated with PMA and ionomycin from WT mice (n = 5/group) and cKO mice (n = 6 for pLIVE group; n = 4 for pLIVE-IL-15 group).

(I) Representative plots showing Annexin V staining (left) and percentage of Annexin V^{+} cells (right) among NK cells in the liver, spleen and lung of pLIVE-IL-15-treated WT (n = 3 for lung and n = 4 for liver and spleen) or cKO mice (n = 4).

Each symbol represents an individual mouse (B–I). Data are the mean ± SEM (ns, not significant; one-way ANOVA (B, C, G, H) or unpaired two-tailed *t*-test (D, E, F, I)). Source data are provided as a Source Data file. Data represent at least two independent experiments (A-I).

Supplementary Figure 9

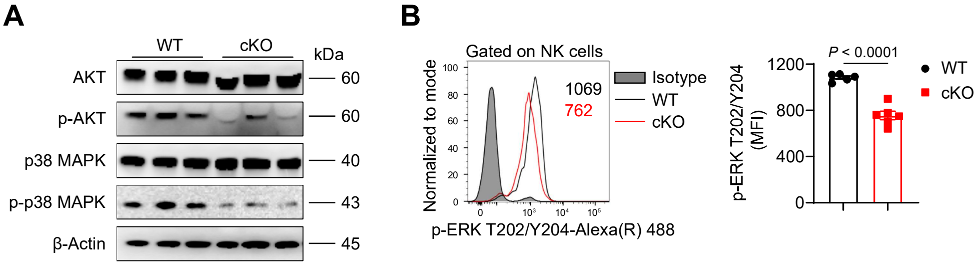

Supplementary Fig. 9

METTL3 is required for sufficient MAPK-ERK activation in NK cells.

(A and B) Splenic NK cells from WT and cKO mice were stimulated with IL-15 (50 ng/mL) for 1 hour in vitro.

(A) Immunoblotting of the indicated molecules in purified splenic NK cells.

(B) Representative histogram (left) and MFI (right) of p-ERK (T202/Y204) expression in splenic NK cells from WT mice (n = 5) or cKO mice (n = 6).

Each symbol represents an individual mouse (B). Data are the mean ± SEM (unpaired two-tailed *t*-test). Source data are provided as a Source Data file. Data represent two independent experiments (A and B).

Supplementary Figure 10

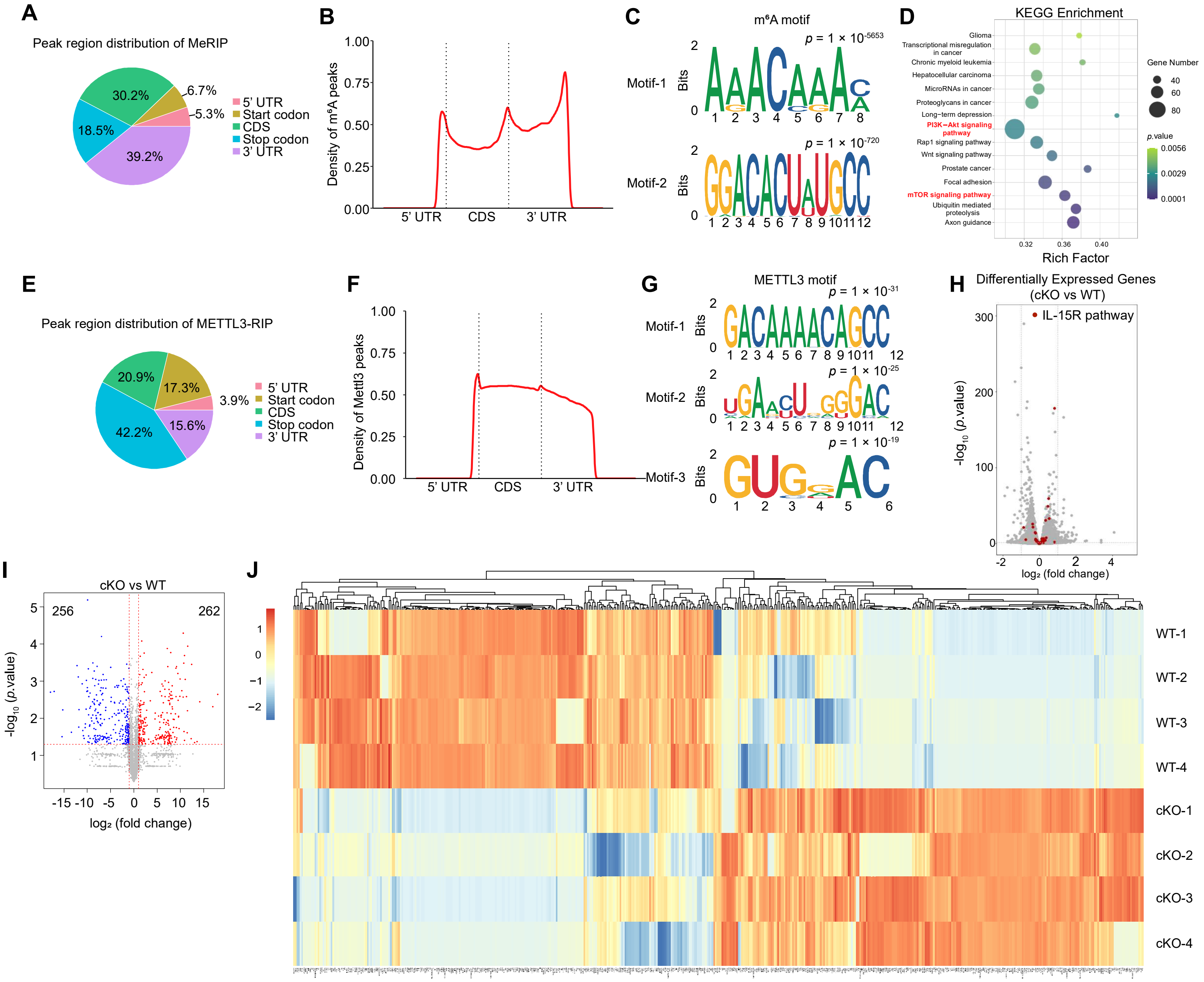

Supplementary Fig. 10

Transcriptome and proteomic analyses of METTL3-deficient NK cells

(A-D) Results of MeRIP-seq of NK cells with two replicates. (A) Venn diagram depicting the percentage of m^6A peak distribution in different transcriptome regions.

(B) Metagene profiles of m^6A peaks across the length of NK cell mRNA.

(C) Sequence motif identified within m^6A peaks of NK cells by HOMER database.

(D) The Kyoto Encyclopedia of Genes and Genomes (KEGG) enrichment analysis of m^6A modified genes (fold enrichment ≥ 5) in NK cells, two-tailed Fisher's exact test, no adjustment was made for *P* value.

(E-G) Results of METTL3-RIP-seq of NK cells with three replicates. (E) Venn diagram depicting the percentage of METTL3 peak distribution in different transcriptome regions.

(F) Metageneprofiles of METTL3-binding peaks across the length of NK cell mRNA.

(G) Sequence motif identified within METTL3-binding peaks of NK cells by HOMER database.

(H) Volcano plot showing the differentially expressed genes by mRNA-seq analysis of splenic NK cells from cKO and WT mice. Red dots represented genes related to IL-15R pathway.

(I) Volcano plot showing the differentially expressed proteins analyzed by mass spectrometry in NK cells of cKO mice, compared with those of WT mice. Blue or red dots indicate proteins downregulated (blue) or upregulated (red) respectively in cKO mice with *P*-value < 0.05 and fold change (KO/WT) < 0.5 or > 2 respectively.

(J) Heatmap plot showing the specific differentially expressed proteins in (I).

Supplementary Table 1. Flow antibodies used in this study

Target	Fluorescence	Reactivity	Source	Clone	Dilution
METTL3	Alexa Fluor 488	Human; Mouse	Abcam	EPR18810	1:500
METTL3	Alexa Fluor 647	Human; Mouse	Abcam	EPR18810	1:500
CD56	BV605	Human	Biolegend	5.1H11	1:200
IFN-γ	APC	Human	BD	4S.B3	1:200
CD45	APC-Cy7	Human	BD	2D1	1:200
NKp30	PE-Cy7	Human	Biolegend	P30-15	1:200
T-bet	PE-Cy7	Human	Biolegend	4B10	1:200
CD69	PE-Cy7	Human	BD	FN50	1:200
NKG2D	PE-Cy7	Human	BD	1D11	1:200
CD3	PerCP-Cy5.5	Human	Biolegend	HIT3a	1:200
7-AAD	--	Mouse	BD	--	1:100
P-mTOR S2448	Alexa Fluor 647	Mouse	BD	021-404	1:25
CD127	BV421	Mouse	BD	A7R34	1:200
Ly49D	BV421	Mouse	BD	4E5	1:200
CD8	BV510	Mouse	BD	53-6.7	1:200
CD27	BV510	Mouse	BD	LG.3A10	1:200
CD45.2	BV786	Mouse	BD	104	1:200
CD3	BV786	Mouse	BD	145-2C11	1:200
IFN-γ	BV786	Mouse	BD	XMG12	1:200
CD3	FITC	Mouse	BD	145-2C11	1:200
CD11c	FITC	Mouse	BD	HL3	1:200
CD69	FITC	Mouse	BD	H1.2F3	1:200
CD107a	FITC	Mouse	BD	1D4B	1:200
Annexin V	FITC	Mouse	BD	--	1:200
NKG2D	PE	Mouse	BD	CX5	1:200
FasL	PE	Mouse	BD	MFL3	1:200
CD122	PE	Mouse	BD	Tm, β1	1:200
2B4	PE	Mouse	BD	2B4	1:200
P-AKT S473	V450	Mouse	BD	M89-61	1:100
Ki67	Alexa Flour 660	Mouse	Thermo	SolA15	1:200
NKG2D	APC	Mouse	Thermo	CX5	1:200
KLRG1	APC	Mouse	Thermo	2F1	1:200
Bcl2	eFluor 450	Mouse	Thermo	10C4	1:200
Eomes	eFluor 450	Mouse	Thermo	Dan11mag	1:200
GzmB	PE	Mouse	Thermo	16G6	1:200
IFN-γ	PE-Cy7	Mouse	Thermo	XMG1.2	1:200
TIGIT	PerCP-eFluor 710	Mouse	Thermo	GIGD7	1:200
Perforin	APC	Mouse	Biolegend	S16009A	1:200
CD4	APC	Mouse	Biolegend	RM44	1:200
CD226	APC	Mouse	Biolegend	10E5	1:200
CD132	APC	Mouse	Biolegend	TUGh4	1:200
CD45.2	APC-Cy7	Mouse	Biolegend	104	1:200
TIGIT	BV421	Mouse	Biolegend	1G9	1:200
TNF	BV421	Mouse	Biolegend	MP6-XT22	1:200
CD11b	BV421	Mouse	Biolegend	M1/70	1:200
NK1.1	BV605	Mouse	Biolegend	PK136	1:200
T-bet	PE	Mouse	Biolegend	4-B10	1:200
CD27	PE	Mouse	Biolegend	LG.3A10	1:200
CD3	PE-Cy7	Mouse	Biolegend	145-2C11	1:200
CD19	PE-Cy7	Mouse	Biolegend	6D5	1:200
Ly49H	PE-Cy7	Mouse	Biolegend	3D10	1:200
NK1.1	PerCP-Cy5.5	Mouse	Biolegend	PK136	1:200
Gr1	PerCP-Cy5.5	Mouse	Biolegend	RB6-8C5	1:200
CD19	PerCP-Cy5.5	Mouse	Biolegend	6D5	1:200
CD11b	PerCP-Cy5.5	Mouse	Biolegend	M1/70	1:200
CD3	PerCP-Cy5.5	Mouse	Biolegend	145-2C11	1:200
Ter119	PerCP-Cy5.5	Mouse	Biolegend	TER119	1:200
P-ERK1/2 (T202/Y204)	Alexa(R) 488	Mouse	Cell Signaling Technology	E10	1:100

Supplementary Table 2. Primers Used for RT-qPCR

Gene Name	Forward Primer (5' to 3')	Reverse Primer (5' to 3')
Il15	TCTCGTGCTACTTGTGTTTCC	TTGGCCTCTGTTTTAGGGAG
Il10	GTCATCGATTTCTCCCCTGTG	ATGGCCTTGTAGACACCTTG
Il27	GTTCAGGGCTATGTCCACAG	AGTCAGGGAAACATTGGGAAG
Il12a	ACAGATGACATGGTGAAGACG	TCGTTCTTGTGTAGTTCAGTG
Il12b	CTTGCAGATGAAGCCTTTGAAG	TGAGGGAGAAGTAGGAATGGG
Il17f	AGGGAAGAAGCAGCCATTG	GCAAGTCCCAACATCAACAG
Ebi3	CTCTTCCTGTCACTTGCCC	GGATACCGAGAAGCATGGC
Il18	CAGCCTGTGTTCGAGGATATG	CACAGCCAGTCCTCTTACTTC
Il17a	AGTCTTTAACTCCCTTGGCG	ATCTATCAGGGTCTTCATTGCG
Il23a	CAGTGTGAAGATGGTTGTGAC	ATGTCAGAGTCAAGCAGGTG
Mettl3	AGAGTGCATGAAAGCCAGTG	TGTTAAGGAAAGAGCAGTCACC
β-Actin	TGACGTTGACATCCGTAAAGACC	CTCAGGAGGAGCAATGATCTTGA

Supplementary Table 3. Gene List Related to IL15R Pathway

Akt1	*Ikzf3*	*Pik3cg*	*Syk*	*Map2k1*
Bad	*Il2ra*	*Pik3r1*	*Pdk1*	*Map2k2*
Bcl2	*Il2rb*	*Ppia*	*Mtor*	*Il15ra*
Cbl	*Il2rg*	*Ptpn6*	*Akt2*	
Cflar	*Irs2*	*Raf1*	*Akt3*	
Crkl	*Jak3*	*Rps6kb1*	*Ptpn11*	
E2f1	*Jak1*	*Shc1*	*Stat3*	
Fas	*Mapk1*	*Socs1*	*Foxc1*	
Fasl	*Mapk3*	*Socs3*	*Gsk3a*	
Fos	*Myc*	*Sos1*	*Gsk3b*	
Grb2	*Nmi*	*Stat5a*	*Grb2*	
Hras	*Pik3ca*	*Stat5b*	*Raf1*	
